# Regulation of harvester ant foraging as a closed-loop excitable system

**DOI:** 10.1371/journal.pcbi.1006200

**Published:** 2018-12-04

**Authors:** Renato Pagliara, Deborah M. Gordon, Naomi Ehrich Leonard

**Affiliations:** 1 Department of Mechanical and Aerospace Engineering, Princeton University, Princeton, New Jersey, United States of America; 2 Department of Biology, Stanford University, Stanford, California, United States of America; Santa Fe Institute, UNITED STATES

## Abstract

Ant colonies regulate activity in response to changing conditions without using centralized control. Desert harvester ant colonies forage for seeds, and regulate foraging to manage a tradeoff between spending and obtaining water. Foragers lose water while outside in the dry air, but ants obtain water by metabolizing the fats in the seeds they eat. Previous work shows that the rate at which an outgoing forager leaves the nest depends on its recent rate of brief antennal contacts with incoming foragers carrying food. We examine how this process can yield foraging rates that are robust to uncertainty and responsive to temperature and humidity across minute-to-hour timescales. To explore possible mechanisms, we develop a low-dimensional analytical model with a small number of parameters that captures observed foraging behavior. The model uses excitability dynamics to represent response to interactions inside the nest and a random delay distribution to represent foraging time outside the nest. We show how feedback from outgoing foragers returning to the nest stabilizes the incoming and outgoing foraging rates to a common value determined by the volatility of available foragers. The model exhibits a critical volatility above which there is sustained foraging at a constant rate and below which foraging stops. To explain how foraging rates adjust to temperature and humidity, we propose that foragers modify their volatility after they leave the nest and become exposed to the environment. Our study highlights the importance of feedback in the regulation of foraging activity and shows how modulation of volatility can explain how foraging activity responds to conditions and varies across colonies. Our model elucidates the role of feedback across many timescales in collective behavior, and may be generalized to other systems driven by excitable dynamics, such as neuronal networks.

## Author summary

We investigate the collective behavior that allows colonies of desert harvester ants to regulate foraging activity in response to environmental conditions. We develop an analytical model connecting three processes: 1) the interactions between foragers returning to the nest and available foragers waiting inside the nest, 2) the effect of these interactions on the likelihood of available foragers to leave the nest to forage, and 3) the return of foragers to the nest after finding seeds. We propose a mechanism in which available foragers modify their response to interactions after their first exposure to the environment. We show how this leads to colony foraging rates that adjust to environmental conditions over time scales from minutes to hours. Our model may prove useful for studying other classes of systems with excitability dynamics that exhibit both stability in behavior and flexibility with respect to environmental conditions.

## Introduction

Social insect colonies operate without central control. Colonies maintain coherence and plasticity in the face of perturbation and change, even though individuals have limited and uncertain information on the state of the group and the state of the environment. Collective behavior emerges from the response of individuals to social interactions and their assessment of the local environment [1–4]. The study of social insects provides opportunities to investigate open, fundamental questions on how collective behavior adjusts to different conditions and how small differences in these adjustments can lead to large differences in behavior across groups.

The regulation of foraging activity in colonies of the harvester ant (*Pogonomyrmex barbatus*) is a well-studied example of collective behavior [5]. Harvester ants live in the hot and dry Southwestern US desert where they forage for seeds scattered by wind and flooding on the timescale of weeks and months. Foragers do not use pheromone trails; instead, they spread out across the foraging area in search of seeds [6]. Thus the regulation of foraging in harvester ant colonies, unlike in honey bees and in ant species that use pheromone trails, does not allocate workers among spatially fixed resources that differ in quality and availability [7–10]. A harvester ant’s foraging trip time may vary with food availability. However, on the scale of the colony’s foraging area, food is not depleted in the course of the foraging period on a given day, and hot and dry conditions, rather than lack of food, can cause colonies to stop foraging.

The regulation of foraging activity manages a tradeoff between spending water and obtaining water and food: foragers lose water while outside in the dry air, but colonies obtain water by metabolizing the fats in the seeds that they eat [11, 12]. Foraging is initiated each morning by a distinct group of workers, the patrollers [13, 14], who leave the nest before the foragers emerge and explore the nest mound and foraging area. It is the safe return of the patrollers that initiates foraging, through encounters inside the nest between foragers and returning patrollers [15]. Once foraging has begun, harvester ant colonies regulate the rate at which foragers leave the nest using the incoming rate of successful foragers returning with food [16–20]. When an ant contacts another ant with its antennae, it perceives the other ant’s cuticular hydrocarbon (CHC) profile [16]. Because conditions outside the nest change the chemistry of the cuticular hydrocarbons, CHC profiles are task-specific [21], so that in the course of antennal contact, one ant can detect whether another is a forager. An available forager, waiting in the entrance chamber inside the nest, is stimulated to leave the nest by antennal contact with foragers carrying food [18–20]. The rate of interactions experienced by an available forager inside the nest entrance chamber correlates with the local density of ants [20]. Thus a higher rate of incoming foragers leads to a higher rate of interactions [19]. Because each forager searches until it finds a seed, the rate of interaction serves as a noisy measurement of the current foraging conditions [6, 22]. A higher rate of forager return, which reflects a greater food supply, increases the likelihood that available foragers will leave the nest to forage [19, 20, 23].

In the integrator model of [20], each available forager inside the nest collects evidence from incoming foragers by integrating its recent experience of antennal contacts. When the integrated stimulus passes a threshold, the available forager is likely to leave the nest; in the absence of interactions the forager is likely to descend from the entrance chamber to the deeper nest [19, 23], protecting the colony from the inherently noisy signal that results from limited and uncertain interactions [24]. The integrator model has been used to study regulation of the outgoing foraging rate on short timescales of minutes [22].

**Fig 1:**
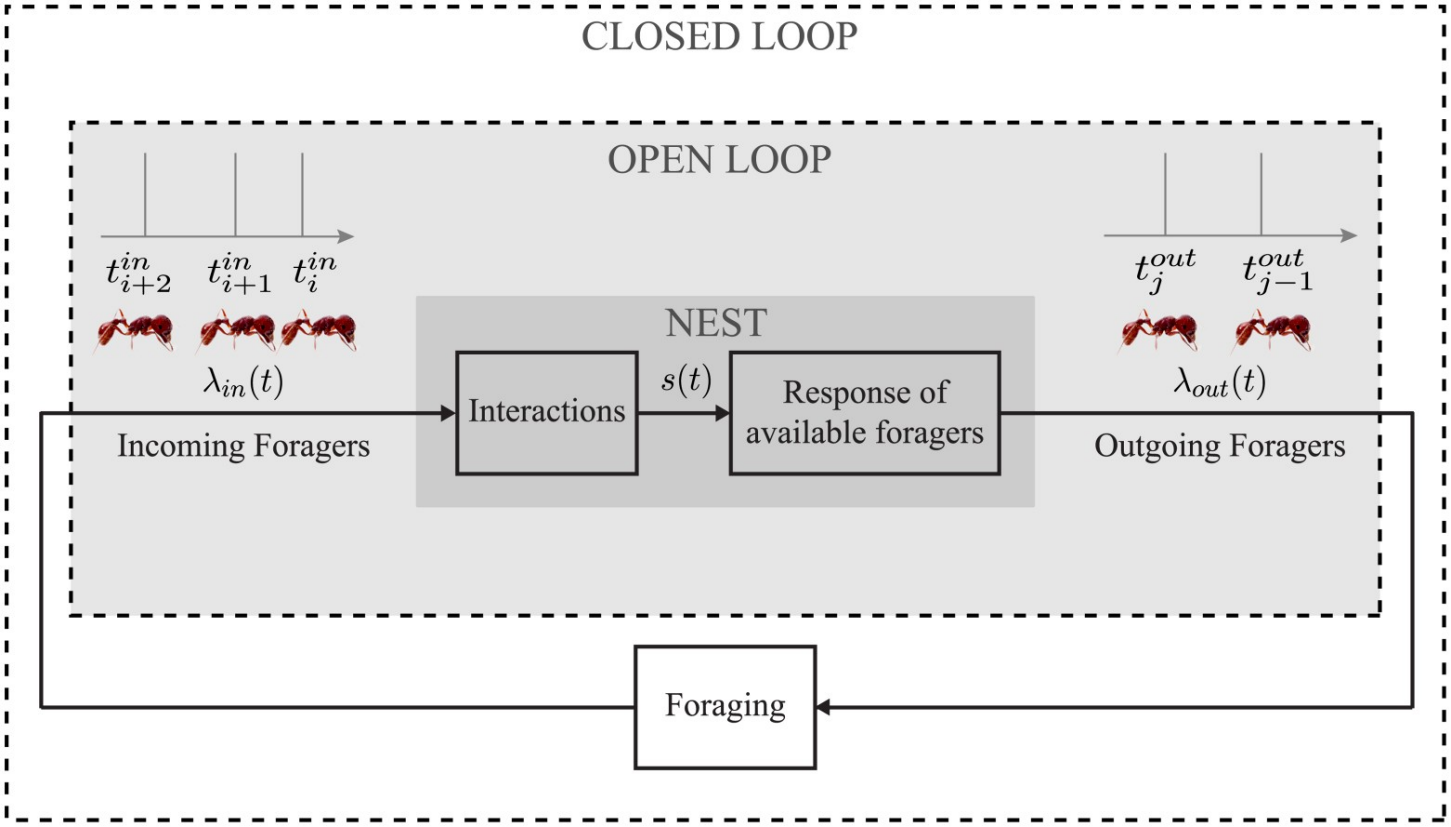
Diagram of the closed-loop model with two components inside the nest and one component outside the nest. The “Interactions” component maps the sequence of incoming foragers λ_*in*_ to a stimulus *s* to represent the result of interactions of available foragers inside the nest entrance chamber with incoming food-bearing foragers. The mapping uses a leaky integrator that increases by a fixed magnitude with every incoming forager and has a natural decay rate. The “Response of available foragers” component maps *s* to the sequence of outgoing foragers λ_*out*_ using the nonlinear FitzHugh-Nagumo oscillator dynamics. Each oscillation represents an ant leaving the nest to forage. The “Foraging” component maps λ_*out*_ to λ_*in*_ using a random time delay with an associated probability distribution to represent the time an ant spends outside the nest foraging.

Colonies regulate their foraging activity on longer timescales, such as from hour to hour, from day to day [25, 26], and across years [5, 25, 27, 28] as colonies grow older and larger. Over timescales from tens of minutes to hours, ants that start as available foragers inside the nest leave the nest to forage, find seeds, return to the nest, and become available foragers again. Thus, the activation of available foragers inside the nest through interactions with incoming foragers is connected in a “closed loop” to the foraging activity outside the nest through feedback of the ants themselves: the stream of foraging ants out of the nest is the input to the foraging activity, and the output of the foraging activity is the stream of foraging ants into the nest (see Fig 1). However, little is known about the role of feedback in the regulation of foraging activity at the timescale of hours and as foraging activity is adjusted to changing environmental conditions. By mid-day in the summer, temperature is high and humidity is low (S1 Fig). Foraging activity increases from its start in early morning and then levels off, often remaining at a steady rate for tens of minutes to hours. It declines to no activity during the heat of the afternoon.

How a colony regulates foraging in response to environmental conditions, especially temperature and humidity, is ecologically important. Colonies live for 20-30 years, the lifetime of the single founding queen who produces short-lived workers year after year. At about five years of age the queen begins to produce reproductives that mate with those of other colonies, and the daughter queens found offspring colonies [29]. Colonies differ in the regulation of foraging and these differences persist from year to year, including variation in how often colonies are active [25] and in how they respond to changing temperature and humidity conditions [5, 26, 28]. The persistence of foraging behavior across years, in subsequent cohorts of workers, and comparison of parent and offspring colonies [29] suggests that foraging behavior is heritable. It appears that colony differences in the regulation of foraging arise from differences in how individuals respond to interactions with incoming foragers, that is, in the rates of interaction required to stimulate a forager to leave the nest [20].

How a colony adjusts foraging activity to low humidity and high temperature is crucial for reproductive success: colonies that conserve water are more likely to have offspring colonies [5]. We hypothesize that these differences among colonies in their response to different environmental conditions are the result of differences in how their foragers assess humidity and in how this influences their response to interactions with incoming foragers [26]. Recent work suggests this depends on variation in the neurophysiology of biogenic amines such as dopamine [30]. Here we model how colonies adjust to environmental conditions by adjusting their “volatility” defined as their sensitivity to interactions with returning foragers. Our goal is to suggest testable hypotheses about the sources of variation among colonies upon which natural selection can act to shape collective behavior.

Previous modeling work has elucidated how the outgoing foraging rate across timescales of minutes depends on the incoming foraging rate [22], and how individuals assess interaction rate [20]. But we do not know how these are combined to adjust foraging activity across minute-to-hour timescales, how steady foraging rates are maintained, how the adjustments may depend on environmental conditions, and how they may differ from colony to colony.

Here we propose a closed-loop model (Fig 1) to address these questions by examining how an incoming forager’s assessment of external conditions provides additional feedback to the colony and in turn adjusts the colony foraging rate. Our model is motivated in part by the frequent use of excitability dynamics to model neurons, and the parallels between ant-to-ant interactions that drive foraging and neuron-to-neuron interactions that underlie the cognitive abilities of organisms [20, 31–34]. Using well-studied excitability dynamics of a weakly interacting collective, we introduce feedback at multiple time-scales and explore general questions concerning stability and responsiveness to a changing environment.

Drawing on theory and tools from dynamical and control systems, we study the relationship, in the model, between the fast activation of foragers inside the nest and the slow feedback from incoming foragers to describe, with a small number of parameters, how the incoming and outgoing foraging rates adjust to changing conditions on the timescale of tens of minutes to hours. We show how the foraging rates are stabilized, and we suggest how small differences in parameter values can lead to variation in the regulation of foraging for different environmental conditions and for different colonies.

## Methods

### Field observations of foraging activity

We performed field observations of red harvester ant colonies at the site of a long term study near Rodeo, New Mexico, USA. Observations were made in August and September of 2015, 2016, and 2017. Foragers leave the nest in streams or trails that can extend up to 20 m from the nest [35]. Each forager leaves the trail to search for seeds, and once it finds food, it returns to the nest [6, 35]. Data on foraging rates were recorded from the beginning of the foraging period in early morning until around noon. We recorded the times at which foragers crossed a line perpendicular to the trail at a distance of about 1 m from the nest entrance, as in previous work (e.g. [22, 28, 36]). The timestamps for each forager crossing the line were recorded either manually in real-time with the assistance of an electronic tablet and custom software, or from video recordings, processed with computer vision software (AnTracks Computer Vision Systems, Mountain View, CA). In some cases we used both tablet and video to ensure that both data collection methods provided similar results.

We denote by $t_{i}^{in}$, i∈N, the sequence of times incoming foragers cross the line and by $t_{j}^{out}$, j∈N, the sequence of times outgoing foragers cross the line. Sequences of incoming and outgoing foragers are represented as sums of infinitesimally narrow, idealized spikes in the form of Dirac-delta functions:

$$\begin{array}{c} \lambda_{in}\left(t\right)=\sum_{i=1}^{n}\delta \left(t-t_{i}^{in}\right),\;\lambda_{out}\left(t\right)=\sum_{j=1}^{m}\delta \left(t-t_{j}^{out}\right), \end{array}$$ (1)

where *n* and *m* are the indices of the last incoming and outgoing forager, respectively, before time *t*. We estimated the instantaneous incoming and outgoing foraging rates, in units of ants/sec, using a sliding window filter with window Δ*t* = 300 sec:

$$\begin{array}{c} r_{in}\left(t\right)=\int_{-\infty }^{\infty }w\left(\zeta \right)\lambda_{in}\left(t-\zeta \right)\;d\zeta ,\;r_{out}\left(t\right)=\int_{-\infty }^{\infty }w\left(\zeta \right)\lambda_{out}\left(t-\zeta \right)\;d\zeta \end{array}$$ (2)

where

$$\begin{array}{c} w\left(t\right)=\{\begin{array}{cc} 1/\Delta t & \text{if}\;-\Delta t/2\leq t\leq \Delta t/2\\ 0 & \text{otherwise}. \end{array} \end{array}$$ (3)

We selected the size of the sliding window to be sufficiently long to remove noise but sufficiently short to preserve the interesting dynamic features of the foraging rates across tens of minutes to hours.

### Model

We propose a low-dimensional dynamic model with a small number of parameters that has sufficiently rich dynamics to capture the range of observed foraging behavior across minute-to-hour timescales and yet retains tractability for analysis. We use the model to systematically investigate the effects of model parameters and environmental conditions, notably temperature and humidity, on foraging rates.

Our model has three components as shown in Fig 1: 1) the *Interactions* component models the accumulation of evidence by available foragers inside the nest entrance chamber from their interactions with incoming foragers carrying food, 2) the *Response of available foragers* component models the activation of available foragers to leave the nest to forage in response to accumulated evidence, and 3) the *Foraging* component models the collecting of seeds outside the nest by active foragers. We assume the total number of foragers *N* (active foragers outside the nest plus available foragers inside the nest) remains constant throughout the foraging day, although this assumption could be relaxed in a generalization of the model.

#### Interactions

We use leaky-integrator dynamics to model the stimulus *s* that the group of available foragers inside the nest entrance chamber experience from their interactions with incoming food-bearing foragers:

$$\begin{array}{c} \frac{ds}{dt}=-\frac{s}{\tau }+k\lambda_{in}. \end{array}$$ (4)

**Fig 2:**
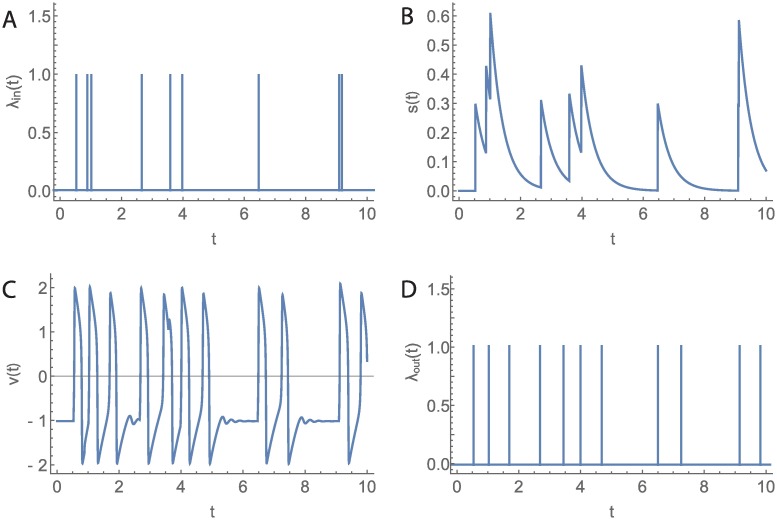
Open-loop model. A) Sequence of incoming foragers λ_*in*_. B) Stimulus signal *s* associated with λ_*in*_. C) FN output *v* for input *s*. D) Sequence of outgoing foragers λ_*out*_ obtained by thresholding FN output from below at 0.75.

The leaky-integrator (4) integrates information from the sequence of incoming foragers (Fig 2A), but the information “leaks,” i.e., decays slowly over time. Here, we use the leaky-integrator dynamics to estimate the instantaneous rate of incoming foragers, which is proportional to the overall rate of interactions experienced by available foragers inside the nest [19]. The continuous-time signal *s* increases by a fixed amount *k* with every incoming forager in λ_*in*_ and decays exponentially back to zero with a time constant of *τ* (Fig 2B).

The leaky-integrator dynamics work as an evidence accumulator that gradually forgets past evidence. These dynamics have been used to model chemical synapses [37] and have been used as the integrate-and-fire neuronal model when there is no reset boundary [38–40].

#### Response of available foragers

We use the FitzHugh-Nagumo (FN) excitability dynamics [41, 42], often used to model neuronal excitability, as a phenomenological model for the activation of available foragers inside the nest entrance chamber. Our choice of dynamics is motivated by the similarities between the ant-to-ant interactions that activate available foragers to leave the nest to forage and the neuron-to-neuron interactions that drive cognition in organisms [20, 31–34]. In the neuronal setting, the FN dynamics model the membrane voltage response of a neuron to an electrical stimulus. The state *v* is the voltage and a second state *u* is a recovery variable that models the flow of ions across the membrane and provides a relatively slow negative feedback on the rate of change of *v*. For low values of stimulus, the voltage remains at rest; for intermediate values, the voltage oscillates; and for large values, the voltage saturates. In our phenomenological model, an oscillation represents the activation of an outgoing forager.

We first consider a homogeneous colony and model the dimensionless, scalar activation state *v* of available foragers in the nest entrance chamber as the fast timescale variable in the FN equations [41, 42]:

$$\begin{array}{c} \epsilon_{1}\epsilon_{2}\frac{dv}{dt}=v-v^{3}/3-cu-a+s \end{array}$$ (5)

$$\begin{array}{c} \epsilon_{1}\frac{du}{dt}=v-cu. \end{array}$$ (6)

These equations describe nonlinear oscillator dynamics with the stimulus *s* of Eq (4) as the input and *v* as the output. Oscillations result from a balance between positive feedback in *v* (first term on the right of Eq (5)) and negative feedback in the dimensionless, slow timescale, recovery variable *u*. The parameter *c*, which scales the negative feedback, modulates the frequency of oscillations and the range of values of stimulus *s* that lead to oscillations. We introduce *c* in both Eqs (5) and (6) so that a change in *c* determines the frequency of oscillations and the range of values of stimulus *s* that lead to oscillations, but it leaves other features of the dynamics unchanged. Since *c* regulates responsiveness, and does so better than parameters *ϵ*_1_, *ϵ*_2_, and *a*, we let *c* represent the *volatility* of the available foragers.

The parameter *ϵ*_2_ defines the time separation between the dynamics of the fast and slow states, and the parameter *ϵ*_1_ defines the time separation between the FN dynamics and the stimulus dynamics (4). The parameter *a* provides an offset to *s* and its value is selected based on the value of *k*, which is the increase in stimulus *s* resulting from an interaction with an incoming forager. Parameter *a* is chosen so that *k* is greater than the threshold above which input *s* elicits an oscillation. Eliciting at least one oscillation per isolated incoming forager in the model allows for a rapid increase in the foraging rates during the first few minutes of foraging, when the initial incoming foraging rate is low.

The activation dynamics (Eqs (5) and (6)) of the available foragers yield three qualitatively distinct dynamical regimes, determined by the magnitude of input *s*, and bifurcation values *b*_1_ and *b*_2_ (Fig 2C). In the first regime, the system remains in a *resting* state for 0 < *s* < *b*_1_. This reflects the situation in which the stimulus for available foragers to leave the nest is low because there are few incoming foragers. In the second regime, which takes place when *b*_1_ < *s* < *b*_2_, the system is in an excited state with oscillations in *v*. This reflects the situation in which incoming foragers are sufficiently frequent to stimulate the available foragers. The transition from resting to oscillatory behavior as *s* increases corresponds to a Hopf bifurcation and *b*_1_ is the corresponding bifurcation point. The oscillations appear as short-lived spikes, and we define each spike for which *v* increases above 0.75 as a forager leaving the nest (Fig 2D). The shortest possible time between foragers leaving the nest is determined by the volatility *c* (see S1 Text).

In the third regime, corresponding to very large values of *s* > *b*_2_, there are no oscillations and the system is fixed in a *saturation* state. The transition from oscillatory to saturated regime is a second Hopf bifurcation with bifurcation point *b*_2_. This means that a high instantaneous incoming rate that produces a high value of *s* will lead to saturation in the FN dynamics resulting in a decrease in instantaneous outgoing rate. Conditions represented by the effect of saturation include 1) overcrowding effects, which reduce the percentage of interactions experienced by each available forager relative to the incoming foraging rate, 2) the limited size of the nest entrance tunnel, which constrains how many foragers can enter and leave the nest in a short amount of time, and 3) the difference in timescales between the high outgoing rates, in seconds, and the time required, in minutes, for foragers to move from the deeper chambers of the nest up to the entrance chamber [19, 23].

#### Foraging

We treat the process of foraging for seeds outside the nest as a random time delay. We model the interval between the time that a forager leaves the nest and the time when it returns with food as a chi-square random variable *X*, with parameter *D* representing the mean foraging time in minutes. The distribution of foraging times *F*(*X*, *D*) is

$$\begin{array}{c} F\left(X,D\right)=1-\frac{\Gamma (D/2,X/2)}{\Gamma (X/2)}, \end{array}$$ (7)

where Γ(*X*) and Γ(*a*, *X*) are the Gamma function and the upper incomplete Gamma function, respectively. This right-skewed distribution is based on field observations of the duration of foraging trips, measured as the total time elapsed from when a forager leaves the nest to when it returns with food [6]. For *D* = 2, *F*(*X*, 2) = 1 − *e*^−*X*/2^.

Our model for the foraging process is equivalent to a queueing system [43] in which arriving customers, represented by outgoing foragers λ_*out*_, find a seed after a given random “service time”. The number of servers in this analogy of the foraging process as a queue is assumed to be infinite because foragers do not need to wait before they start looking for a seed (i.e., before receiving the service). In queueing theory, queues with random service time and infinite number of servers can elucidate the effects of service time on the expected number of customers being serviced at any time.

#### Proposed mechanism for response to environmental conditions

**Fig 3:**
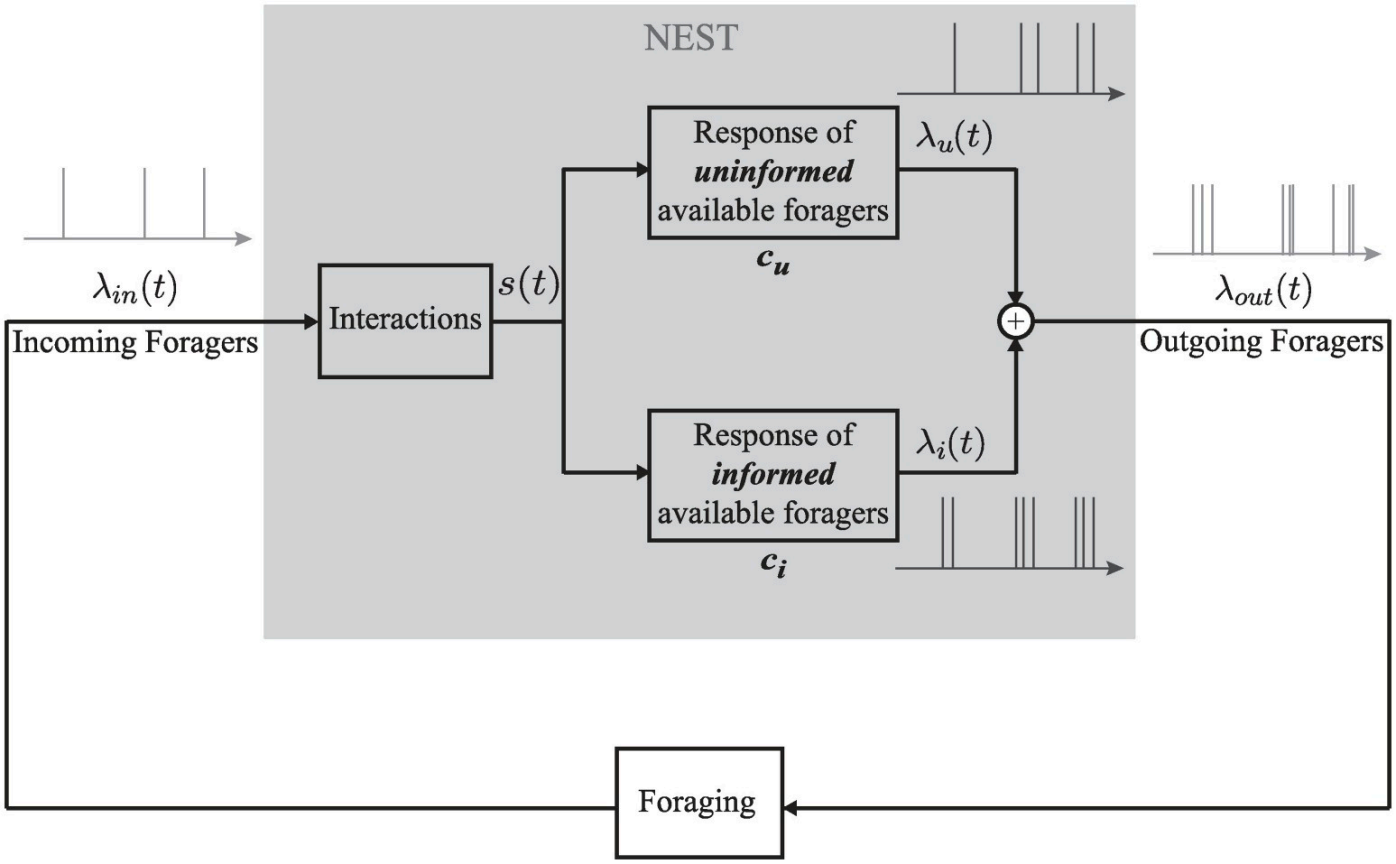
Block diagram of proposed mechanism for response of colony to environmental conditions. The available foragers inside the nest comprise two sets: *f*_*u*_ corresponds to those that have not yet left the nest and so are uninformed about the conditions outside the nest, and *f*_*i*_ corresponds to those informed during a previous foraging trip. The response of each set to *s* is represented by a different FN model, distinguished by the volatility parameter *c*_*u*_ for the uninformed and *c*_*i*_ for the informed. The outputs of these two oscillator dynamics are weighted probabilistically using thinning to get an outgoing stream of foragers λ_*out*_(*t*).

We propose a mechanism for colony response to environmental conditions, illustrated in Fig 3, in which the volatility of a forager changes after it has been on a foraging trip and exposed to the conditions outside the nest. The proposed mechanism is based on measurements showing that the temperature and humidity inside the nest remain constant throughout the foraging activity period (see S1 Fig). This means that foragers have no information about conditions outside until they leave the nest.

As a first approximation, the model changes the volatility of each forager after it leaves the nest to forage for the first time. Available foragers that have not yet been outside the nest, and are therefore uninformed about the current temperature and humidity outside the nest, have volatility *c*_*u*_. Available foragers that have been outside at least once to forage, and are therefore informed about the current temperature and humidity, have volatility *c*_*i*_.

The values of *c*_*u*_ and *c*_*i*_, representing an average uninformed and an average informed available forager, can be any positive real numbers. These values can vary across colonies and across days. The uninformed volatility *c*_*u*_ can vary across days for a given colony, and across colonies, in response to colony needs, such as the amount of brood to be fed and the amount of stored food, colony size, or neurophysiological factors such as biogenic amines (e.g. [30]). The informed volatility *c*_*i*_ reflects response to conditions that change both on the hourly and daily timescales, such as humidity and temperature outside the nest. For example, the hotter and drier it is outside, the smaller the *c*_*i*_, so the foragers become less volatile and thus less likely to make subsequent foraging trips; the cooler and more humid it is outside, the larger the *c*_*i*_, so the foragers become more volatile and thus more likely to make subsequent foraging trips. *c*_*i*_ can vary across colonies due to physiological differences among colonies in response to conditions. There is currently no evidence that *c*_*i*_ depends on how long it takes for a forager to find a seed, but further work is needed to investigate this.

Let *f*_*u*_ be the set of *n*_*u*_ *uninformed* available foragers that have not yet left the nest during the day and thus have no information about the environmental conditions and *f*_*i*_ the set of *n*_*i*_ *informed* available foragers that have been exposed to the environmental conditions during one or more previous foraging trips that day. We assume that once a forager becomes informed, it remains informed for the rest of the foraging day. The ants in *f*_*u*_ have volatility *c*_*u*_ and the ants in *f*_*i*_ have volatility *c*_*i*_. Let *x*_*u*_ = *n*_*u*_/(*n*_*u*_ + *n*_*i*_) and *x*_*i*_ = *n*_*i*_/(*n*_*u*_ + *n*_*i*_) be the fraction of available foragers that are uninformed and informed, respectively, where we assume that *n*_*u*_ + *n*_*i*_ > 0. Then *x*_*u*_ + *x*_*i*_ = 1.

Initially, *x*_*i*_ = 0 and the colony is completely uninformed (*x*_*u*_ = 1). As foragers return to the nest after their first trip, *x*_*i*_ begins to increase and can continue to increase until *x*_*i*_ = 1 (*x*_*u*_ = 0), when all *N* foragers have been outside the nest at least once. How many minutes (or hours) it takes for *x*_*i*_ to transition from 0 to 1 depends on *N*, *D*, and the changing foraging rates. To model the changing foraging rates, we use two sets of FN oscillator dynamics: one to represent the response to *s* of the uninformed ants in *f*_*u*_ with volatility *c*_*u*_ and a second to represent the response to *s* of informed ants in *f*_*i*_ with volatility *c*_*i*_. Let the corresponding sequences of output from the two oscillator dynamics be λ_*u*_ and λ_*i*_, respectively. We define the sequence of outgoing foragers λ_*out*_ as a probabilistic sum of λ_*u*_ and λ_*i*_, using a method called *thinning* [44]: Every event in λ_*i*_ is kept in λ_*out*_ with probability *x*_*i*_, and every event in λ_*u*_ is kept in λ_*out*_ with probability 1 − *x*_*i*_. When *x*_*i*_ = 0 the foraging rate is determined by *c*_*u*_, and when *x*_*i*_ = 1 the foraging rate is determined by *c*_*i*_. When 0 < *x*_*i*_ < 1, the effective *c* will be a nonlinear combination of *c*_*u*_ and *c*_*i*_. The higher the effective *c*, the higher the outgoing foraging rate.

Here foragers adjust their volatility only once after their first foraging trip outside. We find that even with this adjustment at first exposure, the model provides the range of foraging behavior observed. However, the model can be generalized and predictions refined by allowing for adjustments on subsequent foraging trips, and by allowing for other kinds of adjustments. For example, more than two sets of available foragers with different values of volatility can be used to model effects of repeated exposures to the environment, changing conditions on successive trips, or decay of information about the external environment over time. A decrease in *N* (total number of foragers outside and available inside the nest) can be used to model active foragers that return to the deeper nest after exposure to hot and dry outside conditions [19].

## Results

### Observations of regulation of foraging in red harvester ants

Observations of instantaneous foraging rates computed from the 2015, 2016, and 2017 data show that across colonies and days, the incoming and outgoing foraging rates *r*_*in*_(*t*) and *r*_*out*_(*t*), where *t* is time of day, undergo a transient (i.e., a temporary pattern of change) early in the foraging period followed by an equilibration to a near-equal value, i.e., *r*_*in*_(*t*) ≈ *r*_*out*_(*t*), during the middle part of the foraging period.

**Fig 4:**
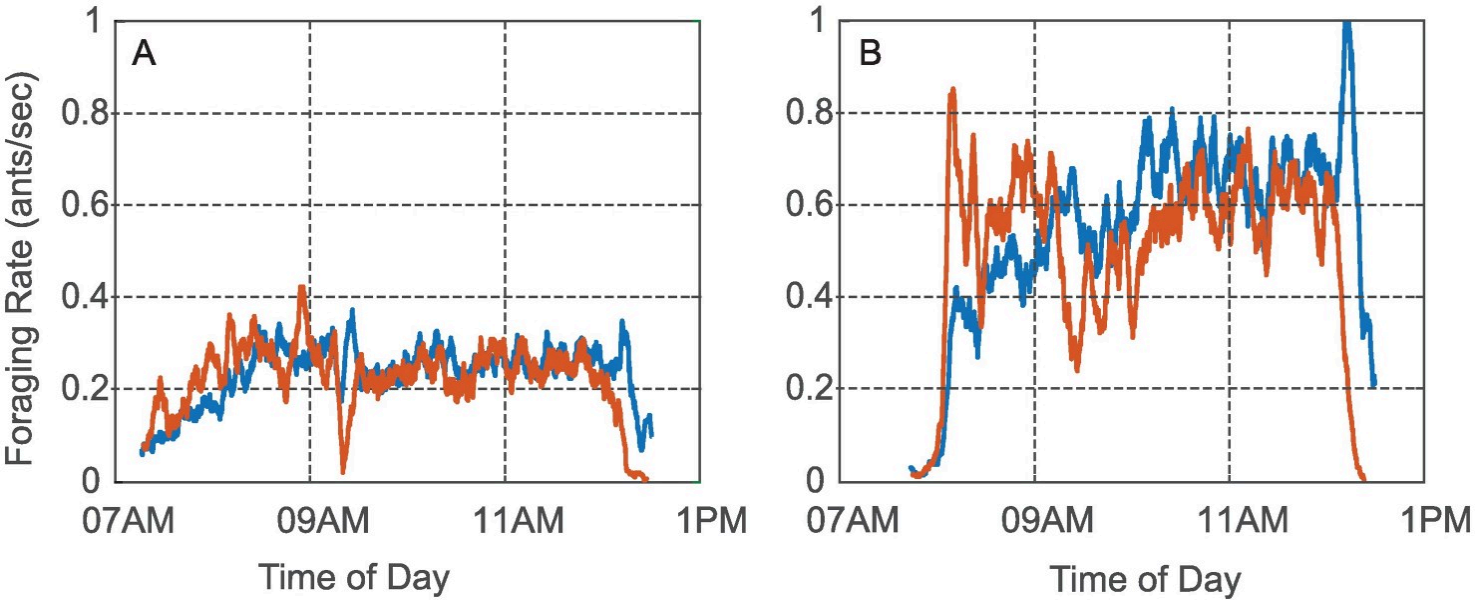
Plots of incoming foraging rate *r*_*in*_ (blue) and outgoing foraging rate *r*_*out*_ (red) versus time of day on August 20, 2016 for A) Colony 1357 and B) Colony 1317. The quasi steady-state (QSS) where incoming and outgoing rates equilibrated to a near-equal value can be observed for both colonies. The QSS rate for Colony 1317 was more than twice as great as it was for Colony 1357.

The equilibration of the incoming and outgoing foraging rates to a near-equal value lasts for intervals from tens of minutes to several hours, and so we refer to it as a quasi steady-state (QSS). We show the data for two colonies in Fig 4. We plot the incoming rate *r*_*in*_ (blue) and the outgoing rate *r*_*out*_ (red) computed from the data for Colony 1357 (Fig 4A) and Colony 1317 (Fig 4B) versus time of day on August 20, 2016. For Colony 1357, the rates equilibrated to a near-equal value early in the day, i.e., between 8:00 and 8:30 am. This was followed by a couple of dynamic adjustments, but then by 9:30 am until just before noon, when all the ants returned to the nest, the incoming and outgoing rates were very closely equilibrated at a QSS rate of around 0.25 ants/sec. Colony 1317 also was observed to reach a QSS. Its incoming and outgoing rates equilibrated to a near equal value shortly after 10:00 am, which lasted until just before noon, when all the ants returned to the nest. Colonies vary greatly in foraging rate [28], and that was true of these two as well. For Colony 1317, the QSS rate was approximately 0.65 ants/sec, more than twice the QSS rate for Colony 1357 on the same day.

**Fig 5:**
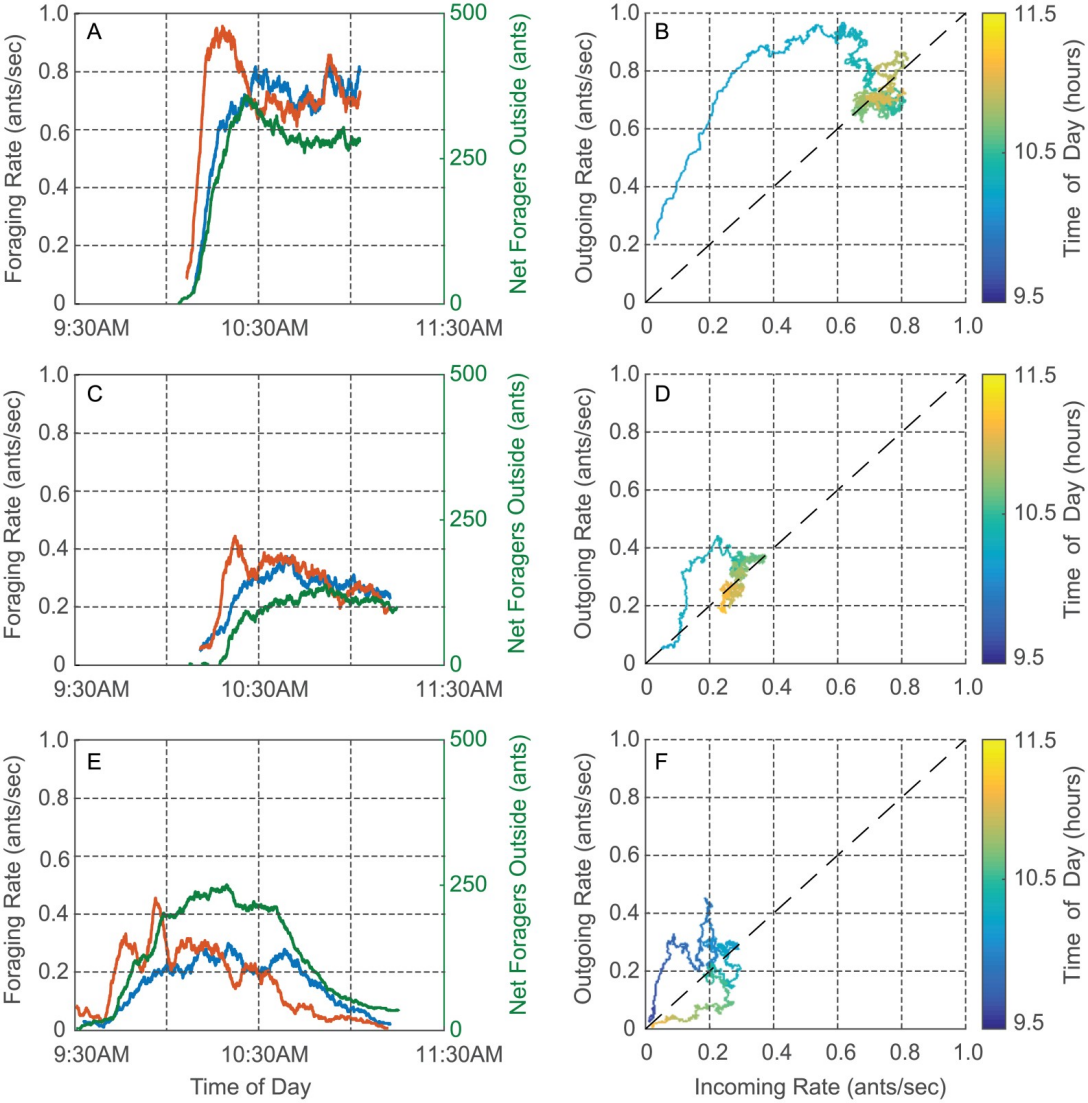
Plots of foraging rate data. Time series plots show incoming foraging rate *r*_*in*_ (blue), outgoing foraging rate *r*_*out*_ (red), and cumulative difference between the number of incoming and outgoing foragers (green) versus time of day. Input-output plots show *r*_*out*_(*t*) versus *r*_*in*_(*t*) with the color scale representing time of day *t*. A) and B) Colony 664 on August 27, 2015. C) and D) Colony 664 on August 31, 2015. E) and F) Colony 863 on September 1, 2015.

We show data for two other colonies in Fig 5. Fig 5A and 5C show *r*_*in*_ (blue) and *r*_*out*_ (red) versus time of day for a single colony, Colony 664, on two different days: August 27, 2015 and August 31, 2015. In each plot, the rates can be seen to come to a near-equal value sometime after 10:30 am. We plot in green the cumulative difference between number of incoming and number of outgoing foragers versus the time of day. The rates are at a QSS when the green curve is approximately horizontal. These data show, as has been observed previously [45], that a given colony varies in foraging rate from day to day, demonstrating that foraging is regulated by processes other than the number of foragers in a colony, which remains relatively constant on the timescale of months. From Fig 5A and 5C it can be seen that Colony 664 reached a QSS rate on August 27, 2015 that is more than twice the QSS rate it reached on August 27, 2015. We note that August 27, 2015 was slightly cooler and more humid than August 31, 2015. On August 27 the average temperature and humidity were 25.3 C and 58% while on August 31 they were 26.8 C and 53%. Moreover, at 11:00 am on August 27, they were 27.5 C and 52% while at 11:00 am on August 31, they were 28.8 C and 45%. Fig 5E shows the data for Colony 863 on September 1, 2015, which were recorded manually. No QSS is observed, i.e., the ants went out but then returned to the nest by 11:00 am without maintaining a steady-state of foragers outside of the nest. Colony 863 did reach a QSS at a reasonably high foraging rate at 11:00 am on September 5, 2015 (see S2A Fig). These observations are consistent with measurements showing that September 1, 2015 was much hotter and drier than September 5, 2015. On September 1 the average temperature and humidity were 25.2 C and 53% while on September 5 they were 22.6 C and 77%. See S1 Table of the SI for more details.

Figs 4 and 5 are representative of observations that suggest the equilibration of incoming and outgoing foraging rates to a near-equal rate to be an important feature in the regulation of foraging in red harvester ant colonies. Further, the equilibrated rate, and the possibility of early cessation of foraging, depend on factors that differ among colonies (Fig 4) and from day to day (Fig 5). We examine the transient in foraging rates further in Fig 5. Early in the foraging day, both *r*_*in*_ and *r*_*out*_ increased rapidly with *r*_*out*_ increasing more rapidly than *r*_*in*_. This led to a rapid increase in the number of active foragers outside the nest. The rapid increase in both rates was followed by a decrease in *r*_*out*_ to the equilibrated near-equal value of the QSS (Fig 5A and 5C) or to an early return of the ants to the nest (Fig 5E).

Input-output plots show the relation between incoming and outgoing foraging rates Fig 5B, 5D and 5F. These figures show the same data as Fig 5A, 5C and 5E, respectively, but plot *r*_*out*_(*t*) versus *r*_*in*_(*t*) with time of day *t* in hours indicated by the color scale. The transient in rates during the early part of the foraging day appear as curved trajectories above the diagonal, because *r*_*out*_(*t*) is typically higher than *r*_*in*_(*t*). In Fig 5B and 5D, the curve rises and then falls to the QSS value where the trajectory then equilibrates around a point on the diagonal corresponding to equal incoming and outgoing rates. This rise and fall of the curve in the input-output plot is typical, even when the trajectory returns to the origin as in the case of Fig 5F.

The data shown in Figs 4 and 5 as well as in S2 Fig are representative of the data collected in 2015, 2016, and 2017. Temperature and humidity for these data sets are given in S1 Table. S2B Fig shows another example of a very early cessation of foraging. S2C and S2D Fig show two different examples of long transients. S2E and S2F Fig show two examples of a burst in the outgoing foraging rate at the start of the foraging day. See S2 Text for details.

### Model dynamics

#### Foraging dynamics inside the nest

Given a sequence of incoming ants λ_*in*_, our open-loop model of foraging dynamics inside the nest (Fig 1) predicts a corresponding sequence of outgoing ants λ_*out*_. We find an analytic approximation for the mapping from mean incoming foraging rate ${\bar{r}}_{in}$ to mean outgoing foraging rate ${\bar{r}}_{out}$, parametrized by volatility *c*. To do so, we assume λ_*in*_ is a Poisson process with (constant) mean incoming rate ${\bar{r}}_{in}$; this is justified for observations of incoming and outgoing sequences of foragers for short periods of time [22].

We assign model parameter values to be *k* = 0.3, *τ* = 0.41, *a* = 0.35, *ϵ*_1_ = 0.2, and *ϵ*_2_ = 0.05, which allow for rich dynamical behavior. While the qualitative behavior is unchanged for different values of *ϵ*_2_ ≪ 1, very high or low values of *a*, *k*, and/or *τ* yield dynamics in which the stimulus *s* is either too low or too high to produce oscillations. So the values for *a*, *k*, and *τ* are selected to balance their opposing effects on *s* and the FN oscillating region.

The oscillating region of the FN dynamics corresponds to the range of values of *s* between the FN bifurcation points *b*_1_ and *b*_2_, computed as $b_{1,2}=a\mp \frac{1}{3}{\left(1-c\epsilon_{2}\right)}^{3/2}$. The offset *a* does not affect the size of the oscillating region whereas the volatility *c* can control it: as *c* increases, the size of the oscillating region decreases (S3 Fig). The offset *a* modifies the lower threshold value of *s*, i.e., the lower bifurcation point *b*_1_, above which the FN oscillates. To ensure that every isolated incoming forager elicits at least one outgoing forager, given *k*, which is the increase in *s* for an interaction with an incoming forager, we choose *a* such that *k* > *b*_1_ for all *c* ∈ [0, 5]. We choose *k* and *τ* to produce sensible values of *s* for the range of incoming foraging rates observed in the data. One of the strengths of the model is that, while there is some flexibility in selecting *a*, *k*, and *τ* such that they satisfy these desired conditions, the qualitative behavior of the system is not affected by the specific values selected.

For very low ${\bar{r}}_{in}$, ${\bar{r}}_{out}$ is low because *s* is low and the FN system remains in the resting state with occasional short-lasting periods of oscillatory behavior (Fig 2). For very high ${\bar{r}}_{in}$, ${\bar{r}}_{out}$ is also low because *s* is high and the FN system remains most of the time in the saturated state. In contrast, ${\bar{r}}_{out}$ is high for ${\bar{r}}_{in}$ that yields an *s* that keeps the FN system inside the oscillating region. In the oscillating region, ${\bar{r}}_{out}$ is equal to the frequency of the oscillations, which is inversely proportional to the volatility *c* as we show in S1 Text.

To get an expression for the natural frequency of the oscillations in the FN, we compute an approximation for its period *T*_*LC*_(*s*, *c*) that uses the time-scale separation between the dynamics of *v* and *u*, see S1 Text and S4 Fig. Under the assumption of a Poisson incoming rate, the process *s* is ergodic (see S3 Text). Thus, over sufficiently long periods of time, suitable time statistics converge to ensemble statistics, allowing us to approximate the fraction of time that *s* spends in the oscillating region using $p(s,{\bar{r}}_{in})$, the probability density function of *s* at steady-state. We compute $p(s,{\bar{r}}_{in})$ in S4 Text as a piecewise function where the piecewise elements satisfy recurrence equations and depend on *k* and *τ*. From this we can construct an analytical expression for ${\bar{r}}_{out}$ as a function of both ${\bar{r}}_{in}$ and *c* (see S3 Text):

$$\begin{array}{c} {\bar{r}}_{out}=\int_{b_{1}\left(c\right)}^{b_{2}\left(c\right)}\frac{p(s,{\bar{r}}_{in})}{T_{LC}\left(s,c\right)}\;ds. \end{array}$$ (8)

**Fig 6:**
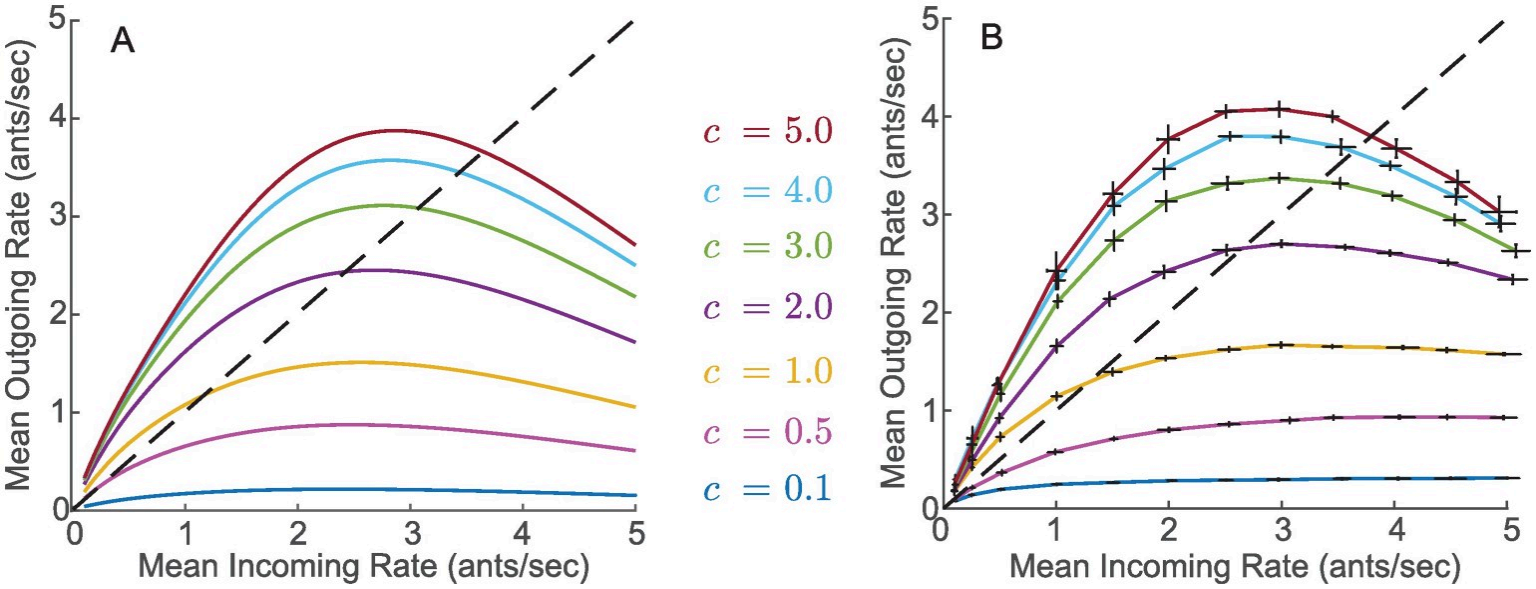
A) Analytical approximations for the nest I/O curves. B) Simulated nest I/O curves for different values of *c*. Each pair of error bars correspond to 10 simulation trials, each 5 minutes long, with a constant expected incoming rate and constant volatility *c*. The dashed black line represents points at which the mean incoming rate ${\bar{r}}_{in}$ is equal to the mean outgoing rate ${\bar{r}}_{out}$.

In Fig 6A we plot ${\bar{r}}_{out}$ versus ${\bar{r}}_{in}$ using Eq (8) for different values of *c*. The resulting open-loop input-output curves, which we call *nest I/O curves* show that the analytic mapping from ${\bar{r}}_{in}$ to ${\bar{r}}_{out}$ depends nonlinearly on *c*. The increasing steepness of the curve at low ${\bar{r}}_{in}$ becomes more pronounced for higher *c* because the frequency of oscillations is proportional to *c*. Similarly, the decreasing steepness of the curves for high ${\bar{r}}_{in}$ also becomes more pronounced for higher *c*. This is because as *c* increases *b*_2_ decreases, causing the FN to saturate at lower ${\bar{r}}_{in}$ values. The maximum value of ${\bar{r}}_{out}$ takes place at the ${\bar{r}}_{in}$ that yields an *s* that keeps the FN system inside the oscillating region. Because of this, the maximum ${\bar{r}}_{out}$ must be less than or equal to the natural frequency of the oscillations at the given value of *c*.

In Fig 6B we show the nest I/O curves obtained by simulating the open-loop system for different constant Poisson incoming rates at a fixed volatility. We measured the resulting mean outgoing rate in each case. We set λ_*in*_ to a five-minutes-long Poisson process, and, in each of 10 simulation trials, we recorded the output λ_*out*_. We computed the mean outgoing foraging rate ${\bar{r}}_{out}$ by dividing the total number of outgoing foragers in the trial by the 300 seconds that the trial lasted. We used the average of the 10 trials as a point estimate for ${\bar{r}}_{out}$ as a function of ${\bar{r}}_{in}$ given the volatility parameter *c*. We constructed nest I/O curves by repeating this point estimation process for twelve different values of ${\bar{r}}_{in}\in \left[0.1,\;5\right]$ while keeping *c* constant.

The simulated I/O curves in Fig 6B are in good agreement with the analytical I/O curves in Fig 6A. The simulation curves are slightly higher than the analytical curves because *T*_*LC*_ underestimates the period of the FN oscillations (see S4 Fig) and because Eq (8) relies on *s* being ergodic (see S3 Text). The fact that there is good agreement between the simulation curves computed from 5-minute-long input sequences and the analytical curves derived under the assumption of an infinite time period suggests that time statistics of *s*(*t*) converge rapidly to ensemble statistics. This rapid convergence indicates that our analytical approximation is also valid across short timescales. We make use of this in our analysis of the closed-loop model dynamics.

The points at which the nest I/O curves in Fig 6 intersect the black dashed diagonal line correspond to ${\bar{r}}_{in}={\bar{r}}_{out}$, which are predictive of the (quasi) steady-state solutions at an equal incoming and outgoing foraging rate observed in the data. Fig 6 suggests that for sufficiently high values of *c*, the equal foraging rate is positive and bounded away from zero, capturing a nontrivial steady-state foraging rate as in Figs 4 and 5B and 5D. However, Fig 6 suggests that for low values of *c*, the equal foraging rate is nearly zero, capturing a steady-state with negligible foraging as in Fig 5F.

To understand how *c* affects the equal foraging rate, consider that, because *k* > *b*_1_, every isolated incoming forager elicits at least one oscillation in the FN output and so at low ${\bar{r}}_{in}$, ${\bar{r}}_{out}$ is equal to or larger than ${\bar{r}}_{in}$. At high *c* values, the frequency of oscillations in the FN is high and an isolated incoming forager will elicit more than one oscillation, resulting in nest I/O curves with an initial slope higher than one and an intersection with the diagonal line at a single point away from the origin. In contrast, for low *c* values, an isolated incoming forager will elicit exactly one oscillation, resulting in nest I/O curves with an initial slope of one, i.e., the curve lies on the diagonal line close to the origin and intersects nowhere else.

This analysis implies the existence of a critical value c* such that if *c* > c*, there is an isolated steady-state solution away from the origin, and if *c* < c*, the steady-state solution remains close to the origin. We find an upper bound $\hat{c}>c^{*}$ so that $c>\hat{c}$ is sufficient for the existence of an isolated steady-state solution away from the origin. For *b*_1_ < *k* < *b*_2_, it can be shown that the number of oscillations caused by a single incoming forager is at most (−*τ* log *b*_1_/*k*)/*T*_*LC*_. We numerically solved this expression for *c* using the asymptotic expansion of *T*_*LC*_ described in S1 Text and found that for $c>\hat{c}=0.5287$ the FN oscillates at least two times per every incoming forager. Therefore, $c>\hat{c}$ is a sufficient condition for the nest I/O curve to lie above the diagonal line at low ${\bar{r}}_{in}$ and to intersect the diagonal line at an isolated point, corresponding to a nontrivial steady-state foraging rate.

#### Foraging dynamics outside the nest

Given a sequence of outgoing foragers λ_*out*_ with rate *r*_*out*_, the foraging dynamics outside the nest predict a corresponding delayed sequence of incoming foragers λ_*in*_ with rate *r*_*in*_. We use results from queueing theory to find analytic expressions relating *r*_*out*_ to *r*_*in*_ and the expected number of active foragers outside the nest.

To facilitate the analysis we assume that λ_*out*_ is a non-homogeneous Poisson process (i.e., a Poisson process with time-varying rate) [22]. Applying known results for queues with a non-homogeneous Poisson distribution of arrival times [46] we obtain the following three results:

1. Let *Q*(*t*) represent the number of active foragers outside the nest, then, for each time *t*′ = *t*/60 where *t* is seconds, *Q*(*t*′) has a Poisson distribution with mean
  $$\begin{array}{c} E\left[Q\left(t^{\prime }\right)\right]=\int_{0}^{\infty }r_{out}\left(t^{\prime }-x\right)\left(1-F\left(x,D\right)\right)\;dx. \end{array}$$ (9)
2. The output process describing how foragers leave the queueing system, that is, the process λ_*in*_ describing how foragers return to the nest, is a non-homogeneous Poisson process with mean
  $$\begin{array}{c} E\left[\lambda_{in}\left(t\right)\right]=\int_{0}^{t}r_{in}\left(x\right)\;dx. \end{array}$$ (10)
3. *r*_*in*_ is related to *r*_*out*_ by
  $$\begin{array}{c} r_{in}\left(t^{\prime }\right)=\int_{0}^{\infty }r_{out}\left(t^{\prime }-x\right)\;dF\left(x,D\right)=E\left[r_{out}\left(t^{\prime }-X\right)\right]. \end{array}$$ (11)

Eqs (7) and (9) show how the number of active foragers outside the nest depends on the history of outgoing foragers. Eq (10) shows that if the outgoing foraging process is a non-homogeneous Poisson process, then the incoming foraging process is also a non-homogeneous Poisson process. And Eq (11) shows how the incoming foraging rate *r*_*in*_ depends on the history of the outgoing foraging rate *r*_*out*_.

At steady-state, the outgoing foraging rate is constant, i.e., *r*_*out*_(*t*) = r*, and Eq (11) reduces to *r*_*in*_ = *r*_*out*_ = r*, i.e., the incoming foraging rate is also constant and equal to the outgoing foraging rate. Moreover, Eq (9) reduces to

$$\begin{array}{c} E\left[Q\right]=r_{out}E\left[X\right]=r^{*}D, \end{array}$$ (12)

i.e., the mean number of active foragers outside the nest is given by the steady-state foraging rate r* multiplied by the average foraging trip time *D*.

The relaxation time for the queue output process to reach steady-state can be analyzed by considering the step-function arrival rate *r*_*out*_(*t*′) = 0 for *t*′ < 0 and *r*_*out*_(*t*′) = r* for *t*′ ≥ 0. Then, from Eq (11), $r_{in}\left(t^{\prime }\right)=r^{*}\int_{0}^{t^{\prime }}dF\left(x,D\right)=r^{*}F\left(t^{\prime },D\right)$ for *t*′ ≥ 0. The difference between the queue input and output rates as a function of time is

$$\begin{array}{c} \left|\right|r_{out}\left(t^{\prime }\right)-r_{in}\left(t^{\prime }\right)\left|\right|=r^{*}\frac{\Gamma (D/2,t^{\prime }/2)}{\Gamma (t^{\prime }/2)} \end{array}$$ (13)

for *t*′ ≥ 0. To illustrate, we compute for *D* = 2 that the right-hand side of Eq (13) simplifies to r* *e*^−*t*′/2^ and the foraging queue converges exponentially in time towards a steady-state where the input and output rates are equal.

#### Closed-loop model dynamics

In our model, outgoing foragers from the nest go out to forage, return to the nest as incoming foragers after finding a seed, and then go back out to forage again if sufficiently excited (Fig 1). Here we show that adding the feedback connection from outgoing to incoming foragers to the open-loop dynamics in the nest yields long-term dynamics with a stable and attracting equilibrium where the incoming and outgoing rates are equal. Stability of an isolated equilibrium implies robustness: the steady-state equilibrated foraging rate is maintained even in the presence of disturbances, e.g., small changes in the rates of incoming foragers. When the volatility *c* > c*, the critical value defined earlier, the steady-state foraging rate is nontrivial, whereas if *c* < c*, the steady-state foraging rate is negligible.

For the dynamics inside the nest, we have shown that *c* parametrizes a family of nest I/O curves, described by Eq (8), which map ${\bar{r}}_{in}$ to ${\bar{r}}_{out}$ across short timescales of a few minutes. For *c* < c*, the nest I/O curve always has slope less than or equal to 1, such that it lies on or below the diagonal line where *r*_*in*_ = *r*_*out*_. For *c* > c*, the nest I/O curve has initial slope greater than 1 and then intersects the diagonal line *r*_*in*_ = *r*_*out*_ at a nontrivial point. For the dynamics outside the nest, we have shown that the mapping from *r*_*out*_(*t*) to *r*_*in*_(*t*) is described across longer timescales of tens of minutes by a time delay given by Eq (11).

We study the closed-loop model dynamics for timescales ranging from tens of minutes to hours by investigating the behavior of a discrete iterated mapping *r*_*n*_ = *G*_*c*_(*r*_*n*−1_) where *r*_*n*_ represents the mean foraging rate at time step *n* and *r*_*n*−1_ represents the mean foraging rate at time step *n* − 1. We can interpret *r*_*n*_ and *r*_*n*−1_ as mean incoming rate or mean outgoing foraging rate since the mean incoming rate becomes the mean outgoing rate after a time delay. The mapping $G_{c}:\;R_{\geq 0}\to R_{\geq 0}$ is defined by the *c*-dependent nest I/O curves shown in Fig 6. *G*_*c*_ describes the process by which the incoming foraging rate becomes the outgoing foraging rate through the dynamics of forager activation inside the nest, which then becomes the incoming foraging rate at a later time.

**Fig 7:**
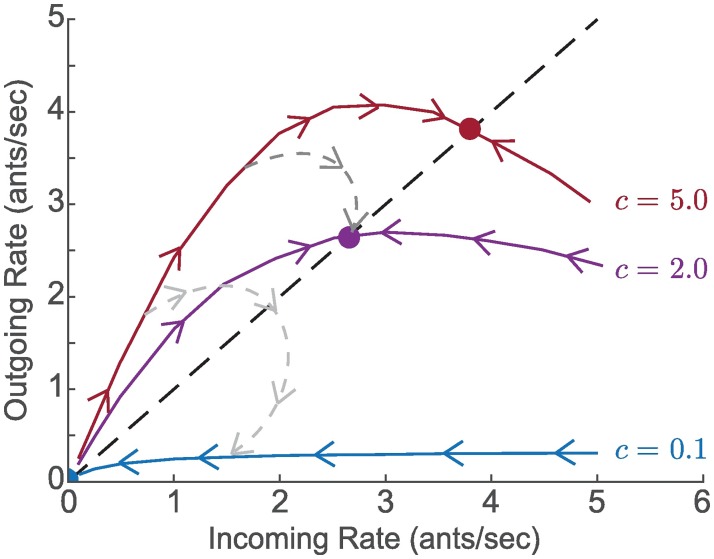
Model dynamics illustrating response of foraging rates to environmental conditions. Red, purple, and blue curves show closed-loop trajectories of *r*_*out*_(*t*) versus *r*_*in*_(*t*) for fixed volatility *c* equal to 5.0, 2.0, and 0.1, respectively. Initially, all available foragers are uninformed about the environment and have volatility *c*_*u*_ = 5.0. The darker gray dashed curve shows the dynamics in the case when foragers exposed to the environment reduce their volatility to *c*_*i*_ = 2.0, as might happen on a moderately hot and dry day. The lighter gray dashed curve shows the dynamics in the case when foragers exposed to the environment reduce their volatility to *c*_*i*_ = 0.1, as might happen on a very hot and dry day.

When *G*_*c*_ lies below (above) the diagonal line where *r*_*in*_ = *r*_*out*_, the average number of outgoing foragers per every incoming forager is less (greater) than one, and iterations of *G*_*c*_ decrease (increase) *r* (Fig 7). For *c* > c*, *G*_*c*_ has one unstable fixed point at the origin and one attractive stable fixed point where *r*_*in*_ = *r*_*out*_. For *c* < c*, *G*_*c*_ has a small interval of fixed points close to the origin. Thus, the closed-loop model dynamics evolve in time towards either a finite steady-state foraging rate *r*_*in*_ = *r*_*out*_ = r* (Fig 7, *c* = 2 and *c* = 5) or to negligible foraging (Fig 7, *c* = 0.1).

**Fig 8:**
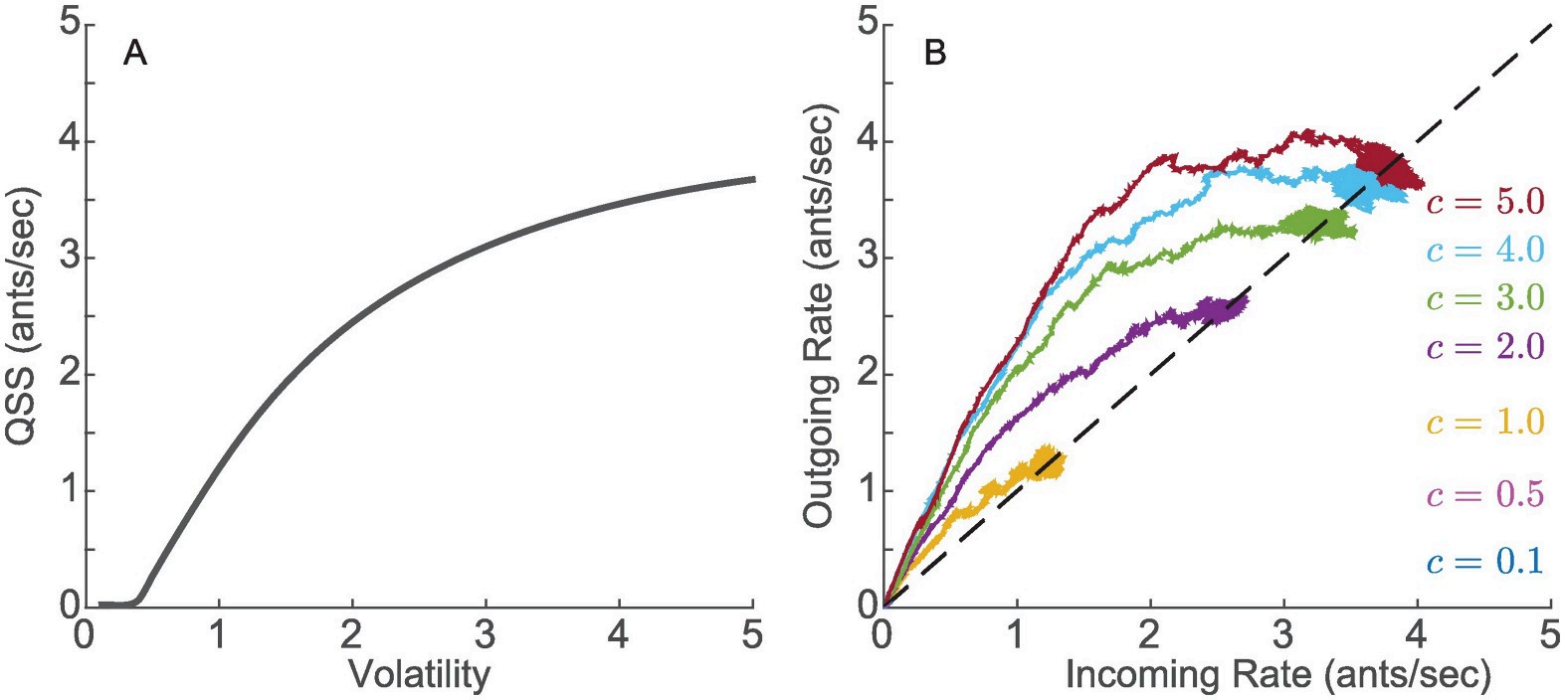
A. Analytical magnitude of the quasi steady-state (QSS) foraging rate obtained from numerically solving Eq (8). B. Closed-loop model simulations for 7 different values of volatility *c*. The initial sequence of incoming foragers for all simulations was set equal to the sequence of incoming foragers recorded during the first 11 minutes for Colony 859 on August 20, 2017, which has a mean incoming rate of 0.01 ants/sec. The total time for all simulations was 3 hours. The mean foraging time was set to 10 minutes (*D* = 10).

The stability of the steady-state equilibrated foraging rate and the implications for robustness result from the balance between positive feedback from incoming ants activating a larger number of outgoing ants, and negative feedback from saturation effects. The magnitude and variance of the steady-state foraging rate increase with *c*. The magnitude also depends on *k* and *τ*, as these affect *s* in Eq (8), which can be numerically solved to find how the magnitude changes with *c* (Fig 8A). We refer to the steady-steady foraging rate as the QSS.

As shown in Fig 8B, simulations of the closed-loop model validate the predictions of the iterated mapping model (Fig 7). We initialize the foraging dynamics by setting λ_*in*_ from *t* = 0 to *t* = 60 × (*D* + 1) seconds to be equal to the initial sequence of incoming foragers for Colony 859 on August 20, 2017, which has the very low mean incoming rate of 0.01 ants/sec during the first 15 minutes (S2C Fig). Using the additional minute, i.e., *D* + 1 instead of *D*, for the initial sequence of incoming foragers helps ensure that the sequence of incoming foragers does not abruptly end before the first few outgoing foragers return to the nest.

#### Closed-loop dynamics with response to environmental conditions

For Poisson sequences of incoming foragers, the mean outgoing foraging rate of the colony is given as the weighted sum of the outputs of the uninformed and informed:

$$\begin{array}{c} {\bar{r}}_{out}=x_{u}\int_{b_{1}\left(c_{u}\right)}^{b_{2}\left(c_{u}\right)}\frac{p(s,{\bar{r}}_{in})}{T_{LC}\left(s,c_{u}\right)}\;ds+x_{i}\int_{b_{1}\left(c_{i}\right)}^{b_{2}\left(c_{i}\right)}\frac{p(s,{\bar{r}}_{in})}{T_{LC}\left(s,c_{i}\right)}\;ds. \end{array}$$ (14)

The closed-loop dynamics can still be studied as an iterated mapping, but we allow the mapping to evolve in time, *G*_*c*_ = *G*_*c*_(*t*), from an initial mapping $G_{c}\left(t_{0}\right)=G_{c_{u}}$ with volatility *c*_*u*_ to a final mapping $G_{c}\left(\infty \right)=G_{c_{i}}$ with volatility *c*_*i*_. The dark and light gray curves in Fig 7 provide an illustration for how the map *G*_*c*_(*t*) changes with time when *c*_*u*_ = 5.0, and *c*_*i*_ = 2.0 or *c*_*i*_ = 0.1. The dynamics first evolve along $G_{c_{u}}$ (red), but as *x*_*i*_ increases, the dynamics shift increasingly to $G_{c_{i}}$, and the trajectory on the plot of *r*_*out*_(*t*) versus *r*_*in*_(*t*) moves towards the *c*_*i*_ curve. In the case *c*_*i*_ = 2.0, the trajectory converges to the fixed point associated with *c* = 2.0 (darker gray dashed curve). In the case *c*_*i*_ = 0.1, the trajectory converges to the only fixed point of $G_{c_{i}}$, which is the origin, leading to a cessation of foraging (lighter gray dashed curve).

**Fig 9:**
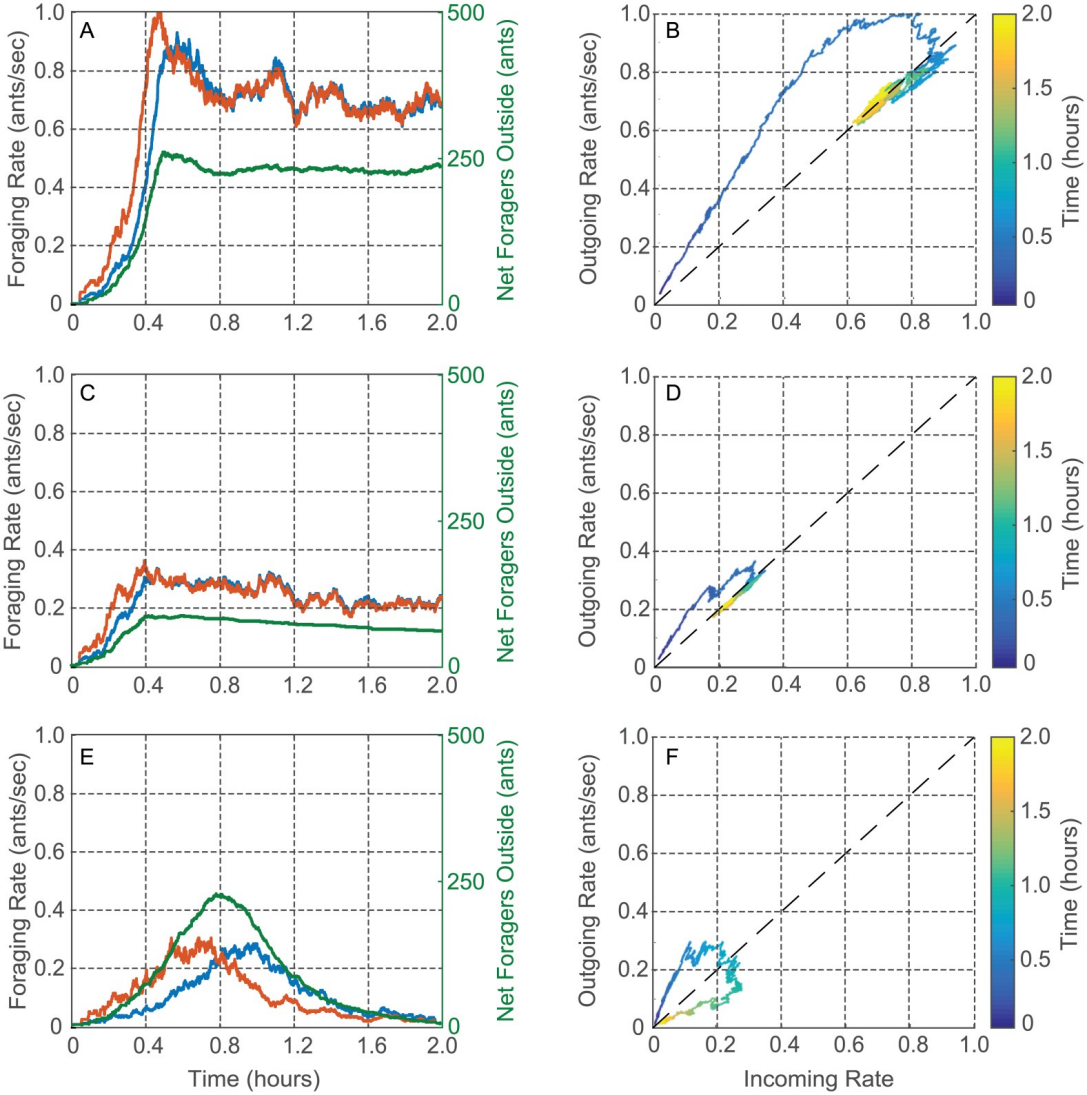
Simulations of the closed-loop model with the adaptation mechanism. Plots are of the same form as in Fig 5, and qualitative comparisons can be made between A and B here and Fig 5A and 5B, between C and D here and Fig 5C and 5D, and between E and F here and Fig 5E and 5F. A) and B) *c*_*u*_ = 3, *c*_*i*_ = 0.9, *N* = 500, *D* = 5. C) and D) *c*_*u*_ = 3, *c*_*i*_ = 0.75, *N* = 200, *D* = 5. E) and F) *c*_*u*_ = 5, *c*_*i*_ = 0.02, *N* = 600, *D* = 15.

Fig 9 shows the resulting time-series and input-output plots for three different simulations of the model with the mechanism for response to environmental conditions. The simulations are distinguished by the set of four parameters: *c*_*u*_, *c*_*i*_, *N*, and *D*. The simulated trajectories qualitatively resemble the trajectories from the field observations shown in Fig 5. We set the initial sequence of incoming foragers as in Fig 8B.

Fig 9A and 9B show the results for *c*_*u*_ = 3, *c*_*i*_ = 0.9, *N* = 500, and *D* = 5. In this case, *c*_*u*_ is much higher than *c*_*i*_, leading to a system with an overshoot behavior in which the outgoing foraging rate increases more rapidly than the incoming rate and then decreases before settling around a steady-state where the rates are approximately equal to 0.7 ants/sec. This is qualitatively similar to the observations of Colony 664 on August 27, 2015 of Fig 5A and 5B. The result of a relatively small number of total foragers *N* and short mean foraging time *D* is that the fraction of informed foragers increases rapidly, leading to a quick convergence towards the steady-state. The net number of foragers outside the nest at steady-state fluctuates with low variability at around 230, close to the prediction given by Eq (12).

Fig 9C and 9D show the results for *c*_*u*_ = 3, *c*_*i*_ = 0.75, *N* = 200, and *D* = 5. This case simulates the same colony as in Fig 9A and 9B but on a hotter and drier day, when the total number of ants *N* that engage in foraging may be reduced and the volatility of the informed ants *c*_*i*_ may be reduced. The overshoot behavior is followed by the foraging rates settling around a steady-state of about 0.25 ants/sec. The result of low values of *N* and *D* is that the fraction of informed foragers increases very rapidly, leading to a rapid convergence towards the steady-state. This is qualitatively similar to the observations of Colony 664 on August 31, 2015 of Fig 5C and 5D.

Fig 9E and 9F show the simulation results for *c*_*u*_ = 5, *c*_*i*_ = 0.02, *N* = 600, *D* = 15. In this case, *c*_*i*_ is close to zero, leading to a colony that goes out to forage but then returns to the nest without sustained foraging. The result of the long mean foraging time *D* is that the fraction of informed foragers increases at a slow rate, leading to longer lasting transient dynamics towards the steady-state. This is qualitatively similar to the observations of Colony 664 on August 27, 2015 of Fig 5E and 5F.

The time it takes for the colony to transition from fully uninformed to fully informed about outside conditions is dictated by *c*_*u*_, *c*_*i*_, *D*, *N*, and the initial conditions for *r*_*in*_ and *r*_*out*_. Low values of *c*_*u*_ result in initially low outgoing foraging rates, so that the corresponding rate at which foragers become informed is low too, even if *c*_*i*_ is high (S5A Fig). Low values of *c*_*i*_ can cause long transients, because once a critical number of foragers has become informed, low volatility makes it difficult for the remaining foragers to become informed. High *D* and *N* can also result in long transients because the time it takes for the transition to a fully informed state depends on the number of available foragers and on how long it takes for informed foragers to return to the nest (S5B Fig). Finally, initially high values of *r*_*out*_ produce a rapidly increasing number of active foragers, reducing the time it takes to reach the informed state with foraging rates that reach a QSS (S5C Fig). Qualitative comparisons can be made between the simulations in S5A, S5B and S5C Fig, and the data in S2C, S2D, S2E and S2F Fig, respectively.

## Discussion

We have derived and analyzed a low-dimensional analytical model of foraging dynamics that requires only a small number of parameters to qualitatively capture a wide range of transient and steady-state features observed in the foraging rates of red harvester ant colonies. Our model extends previous work by using feedback at multiple timescales to account for how foraging rates to and from the nest change over long timescales, from tens of minutes to hours.

Importantly, the long timescales allow for a model-based investigation into how a colony, with no centralized control and little individual information about the state of the colony or environment, can stably regulate its foraging rates and be responsive to temperature and humidity outside the nest across minute-to-hour timescales. Stability implies robustness of the steady-state foraging rate to small disturbances, e.g., small changes in the rate of incoming foragers. Further, because the model is analytically tractable, it can be used to systematically derive empirically testable predictions of foraging behavior as a function of critical model parameters, including number of foragers *N*, mean foraging trip time *D*, and volatility *c*. In our model, these parameters determine the steady-state foraging rate, independent of initial foraging rates. The transient and convergence time to the steady-state, however, do depend on initial rates; higher initial rates lead to faster convergence. The model suggests that a change in volatility as the foragers become exposed to the temperature and humidity outside the nest can account for the observed foraging behavior under different environmental conditions. Further, the model suggests that differences among colonies in volatility, in response to temperature and humidity, can produce the observed variation among colonies in the regulation of foraging.

Our model and analysis highlight the importance of feedback across multiple timescales in the regulation of foraging activity. Previous work isolates the open-loop dynamics inside the nest, which maps incoming ants to outgoing ants on very short timescales. We address the minute-to-hour timescales by examining analytically the closed-loop dynamics that connect the slow foraging activity outside the nest to the fast activation of foragers inside the nest through feedback generated by the ants themselves and their interactions with others. The stream of foraging ants out of the nest is the input to the foraging activity, and the output of the foraging activity is the stream of foraging ants into the nest. The effective volatility of the colony also changes in the model at the timescale of minutes to hours, as foragers leave the nest for the first time and become exposed to the outside humidity and temperature, yielding flexibility in the regulation of foraging activity at minute-to-hour timescales.

In the model, volatility *c* approximates the average sensitivity of available foragers in the nest to interactions with incoming foragers: the higher the *c* the fewer interactions needed to activate available foragers to go out and forage. The relationship between *c* and the activation of foragers is nonlinear, and the subtleties of our model reflect some of the complexities of the system. We use analytical predictions to show how *c* determines three important features of the foraging model dynamics (see Fig 8): 1) the initial transient in incoming and outgoing foraging rates, parametrized by *c*, 2) the equilibration of incoming and outgoing foraging rates to a stable, and thus robust, quasi steady-state rate, parametrized by *c*, and 3) the prediction of an early cessation of foraging or no foraging at all if *c* < c*, a critical volatility value c*.

The behavior of different colonies on the same day or the same colony on different days correspond in the model to different values of *c*. Lower values of *c* result in model dynamics that are consistent with data for hotter and drier days, because lower *c* means that available foragers are less volatile and thus less likely to go out and forage. Higher values of *c* result in model dynamics that are consistent with data for cooler and more humid days, because higher *c* means that available foragers are more volatile and thus more likely to go out and forage. The model distinguishes the volatility *c*_*u*_ of available foragers in the nest who have yet to to go on a foraging trip from the volatility *c*_*i*_ of available foragers in the nest who have already been outside the nest and been exposed to the environment (Fig 3). The result is a transition from the foraging activity of ants with volatility *c*_*u*_ to the foraging activity of ants with volatility *c*_*i*_, which can last from minutes to hours as each of the total *N* ants goes out at a different time on its first foraging trip and returns to the nest after foraging for an average of *D* minutes (Fig 7).

Differences among colonies in the values of parameters *c*_*u*_, *c*_*i*_, *N*, and *D* could lead to the differences among colonies in foraging behavior that we observe. Indeed, over a range of values for the four parameters *c*_*u*_, *c*_*i*_, *N*, and *D*, the model describes the range of transient and quasi steady-state foraging rate behavior observed in the data collected for red harvester colonies in August and September of 2015, 2016, and 2017. The model thus suggests hypotheses about the physiological processes that would lead to different parameter values, such as differences among colonies in how outgoing foragers respond to interactions with returning foragers, and differences among colonies in how foragers respond to conditions such as humidity.

The model represents the case in which foragers make an adjustment to their volatility only after their first foraging trip. To include more variability within a colony the model could be generalized to *M* > 2 groups of available foragers in the nest, distinguished by *M* values of volatility *c*_1_, …, *c*_*M*_. For example, the generalization could be used to account for foragers that make adjustments to how they respond to interactions in the nest after subsequent foraging trips due to repeated exposure or changing temperature and humidity. The generalization could also be used to account for decay of information for those foragers who stay in the nest for a long period after a foraging trip, or to represent foragers that return to the deeper nest after exposure to hot and dry outside conditions.

Foraging models that consider the regulation of foraging activity tend to fall into two categories: multi-agent models that keep track of every individual [7–9], and compartmental models that keep track of the time evolution of fractions of individuals engaged in a specific task [10, 47]. Multi-agent models allow for a detailed modeling of foraging dynamics, often relying on simulations due to their complexity and poor analytical tractability. In contrast, compartmental models provide high tractability in many cases but assume very large group sizes which affect predictions when the group size is small. Our model considers two idealized processes, the activation of foragers inside the nest and the collection of seeds outside the nest, to generate a dynamical system with a small number of equations and parameters. The model accommodates any group size and retains sufficient tractability to generate predictions on the impact of critical model parameters.

Our biologically informed, low-dimensional, and simply parameterized model allows for systematic exploration of mechanisms and sensitivities that can explain collective behavior and guide further theoretical and experimental investigations. Our use of well-studied excitability dynamics opens the way for comparison with other complex systems, such as neuronal networks, that are driven by excitable dynamics. The model together with our analysis based on dynamics and control theory contribute to a better understanding of the role of feedback across multiple timescales in collective behavior.

## Supporting information

S1 Fig Humidity and temperature at surface of desert soil and inside the nest entrance chamber. Humidity and temperature readings recorded on the surface of the desert soil (blue) and inside the nest entrance chamber (red). Temperature and humidity ibutton sensors were placed outside but close to the nest entrance on the desert soil, unshaded, and inside in the nest in an excavated hole, which had been uncovered by excavation and then covered with glass on top and shaded. The humidity and temperature outside the nest changed significantly throughout the morning hours while the humidity and temperature inside the nest entrance chamber remained relatively constant. The measured moderate rise in temperature inside the nest is likely due to the light coming into the nest entrance chamber through the glass. A) Humidity on August 29, 2014 (Colony E). B) Temperature on August 29, 2014 (Colony E). C) Humidity on August 31, 2015 (Colony 10). D) Temperature on August 31, 2015 (Colony 10). E) Humidity on September 1, 2015 (Colony 10). F) Temperature on September 1, 2015 (Colony 10).
(TIF)

S2 Fig Additional field observations of foraging rates. Incoming foraging rate *r*_*in*_ (blue), outgoing foraging rate *r*_*out*_ (red), and difference between number of incoming and outgoing foragers (green) versus time of day. A) Colony 863 September 5, 2015 reached a QSS at a high rate; compare to Fig 4E when on the much hotter and drier day, September 1, 2015, Colony 863 returned to the nest early. B) Colony D19 August 08, 2016 returned to the nest early; the day was very hot and dry. C) Colony 859 August 20, 2017; the transient started late in the morning. The day was cool and humid. D) Colony 1107 August 16, 2017; the transient was slow. The day was dry. E) Colony 1017 August 23, 2016; the initial transient was more like a burst of outgoing foragers. The day was dry. F) Colony 1015 August 18, 2016; another initial burst of outgoing foragers. The day was very dry.
(TIF)

S3 Fig Probability density function for the stimulus function. Each curve represents the PDF *p* of the stimulus function *s* for different values of incoming rate *r*_*in*_. The gray rectangles represent the size of the oscillatory region in the FN system (*b*_1_, *b*_2_) for *a* = 0.35 and different values of volatility *c*. For all curves, *k* = 0.3, *τ* = 0.41.
(TIF)

S4 Fig Period of FN limit cycle when s = 0.35. Blue dots represent numerical simulations for the period of the FN limit cycle. The red curve represents the analytical approximation in S1 Text. In both cases we set *s* = 0.35.
(TIF)

S5 Fig Additional simulations of the closed-loop system with the adaptation mechanism. Plots resemble observed foraging behaviors in S2 Fig. Qualitative comparisons can be made between A here and S2C Fig, between B here and S2D Fig, and between C here and in S2E and S2F Fig. A) *c*_*u*_ = 0.9, *c*_*i*_ = 2.2, *N* = 500, *D* = 5. Setting *c*_*u*_ < *c*_*i*_ where *c*_*u*_ is close to c* results in a long period before the rates ramp up. B) *c*_*u*_ = 1, *c*_*i*_ = 1, *N* = 1000, *D* = 15. Setting the mean foraging trip time *D* to be large results in long lasting transients. C) *c*_*u*_ = 0.7, *c*_*i*_ = 0.9, *N* = 1000, *D* = 7. Setting the initial λ_*in*_ equal to the sequence from the first 5 minutes of λ_*in*_ for Colony 1017 on Aug. 23, 2016 yields the behavior shown in S2E and S2F Fig that follows an initial burst of foragers.
(TIF)

S1 Table Temperature and relative humidity in Rodeo, New Mexico. Average temperature, average relative humidity, temperature at 11 am, and relative humidity at 11 am in Rodeo, New Mexico, USA for days with data plotted in Figs 3 and 4 and S2 Fig. Data collected by the Citizen Weather Observer Program station E8703 and accessed through Weather Underground. The station is located 1.7 miles from the study site.
(PDF)

S1 Text Effect of volatility on the frequency of oscillations in the FN. (PDF)

S2 Text Additional field observations of foraging rates. (PDF)

S3 TextAnalytical approximation for ${\bar{\text{r}}}_{\text{out}}$ in terms of ${\bar{\text{r}}}_{\text{in}}$ and c.(PDF)

S4 Text Probability density function of s(t). (PDF)
