# Supplementary figures and images for: Regulation of harvester ant foraging as a closed-loop excitable system
Source: PLoS Comput Biol. 2018 Dec 4;14(12):e1006200. doi: 10.1371/journal.pcbi.1006200 (PMC6294393; doi:10.1371/journal.pcbi.1006200)

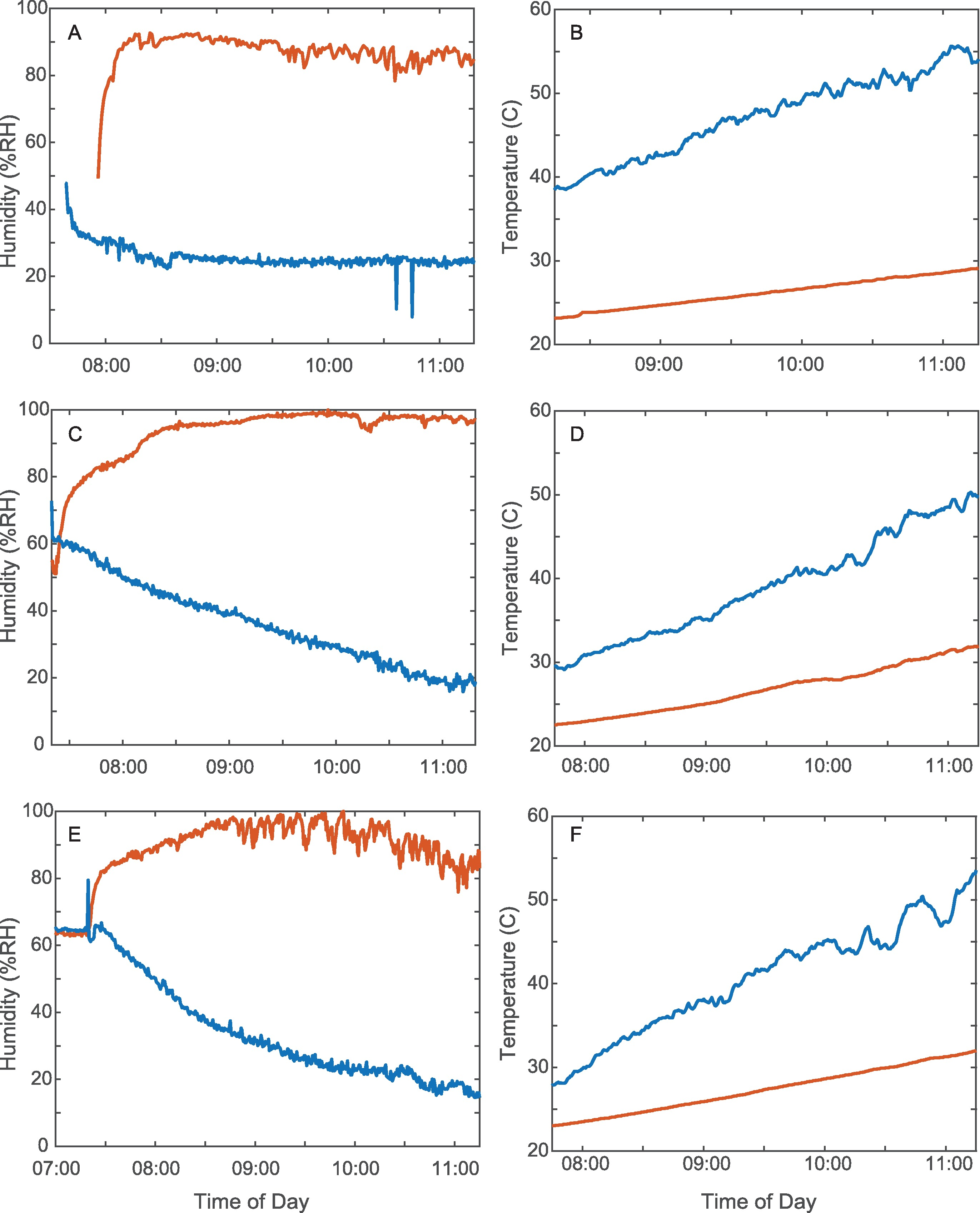

Supplement: S1 Fig — Humidity and temperature readings recorded on the surface of the desert soil (blue) and inside the nest entrance chamber (red). Temperature and humidity ibutton sensors were placed outside but close to the nest entrance on the desert soil, unshaded, and inside in the nest in an excavated hole, which had been uncovered by excavation and then covered with glass on top and shaded. The humidity and temperature outside the nest changed significantly throughout the morning hours while the humidity and temperature inside the nest entrance chamber remained relatively constant. The measured moderate rise in temperature inside the nest is likely due to the light coming into the nest entrance chamber through the glass. A) Humidity on August 29, 2014 (Colony E). B) Temperature on August 29, 2014 (Colony E). C) Humidity on August 31, 2015 (Colony 10). D) Temperature on August 31, 2015 (Colony 10). E) Humidity on September 1, 2015 (Colony 10). F) Temperature on September 1, 2015 (Colony 10). (TIF) [file pcbi.1006200.s001.tif]

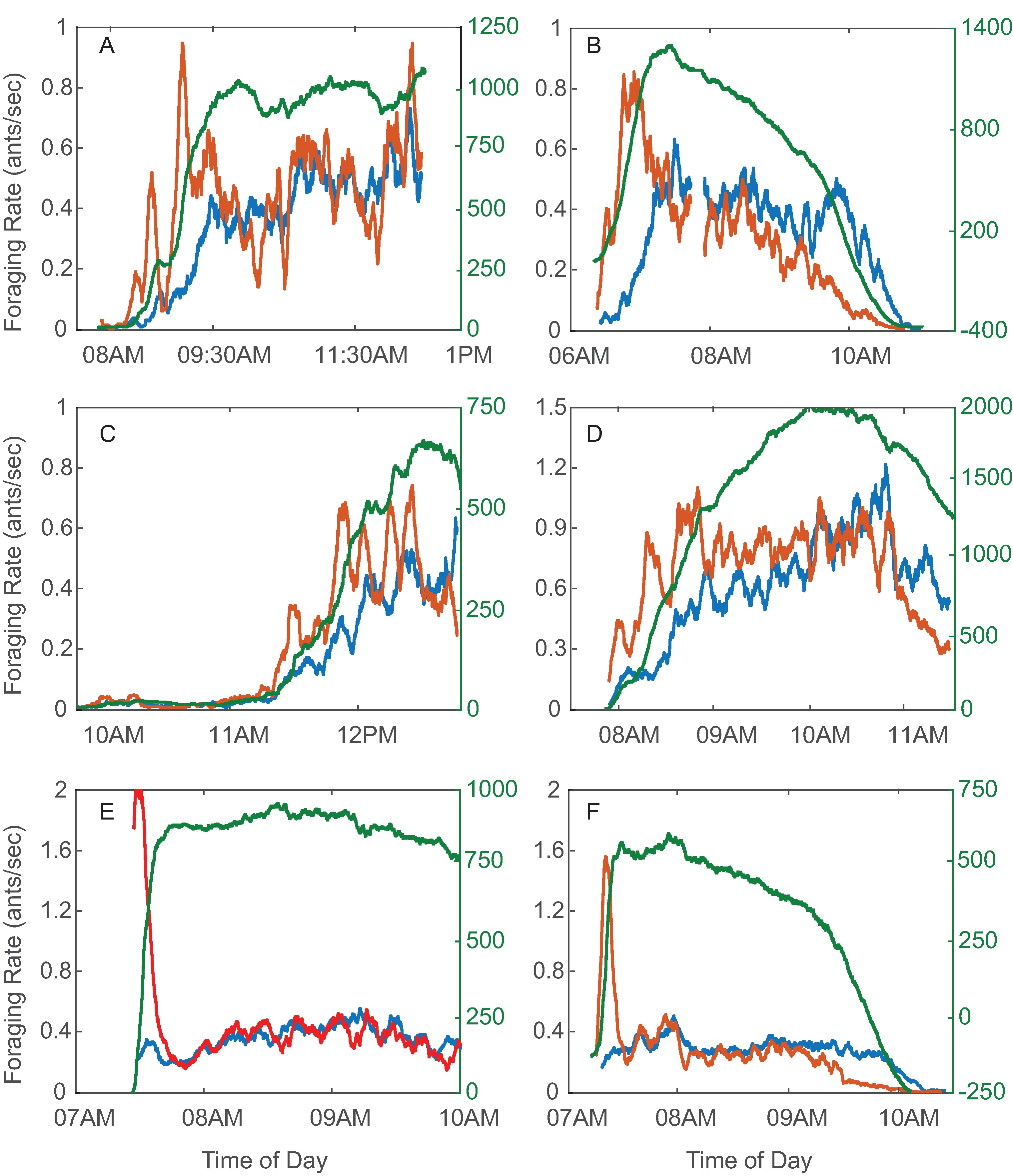

Supplement: S2 Fig — Incoming foraging rate rin (blue), outgoing foraging rate rout (red), and difference between number of incoming and outgoing foragers (green) versus time of day. A) Colony 863 September 5, 2015 reached a QSS at a high rate; compare to Fig 4E when on the much hotter and drier day, September 1, 2015, Colony 863 returned to the nest early. B) Colony D19 August 08, 2016 returned to the nest early; the day was very hot and dry. C) Colony 859 August 20, 2017; the transient started late in the morning. The day was cool and humid. D) Colony 1107 August 16, 2017; the transient was slow. The day was dry. E) Colony 1017 August 23, 2016; the initial transient was more like a burst of outgoing foragers. The day was dry. F) Colony 1015 August 18, 2016; another initial burst of outgoing foragers. The day was very dry. (TIF) [file pcbi.1006200.s002.tif]

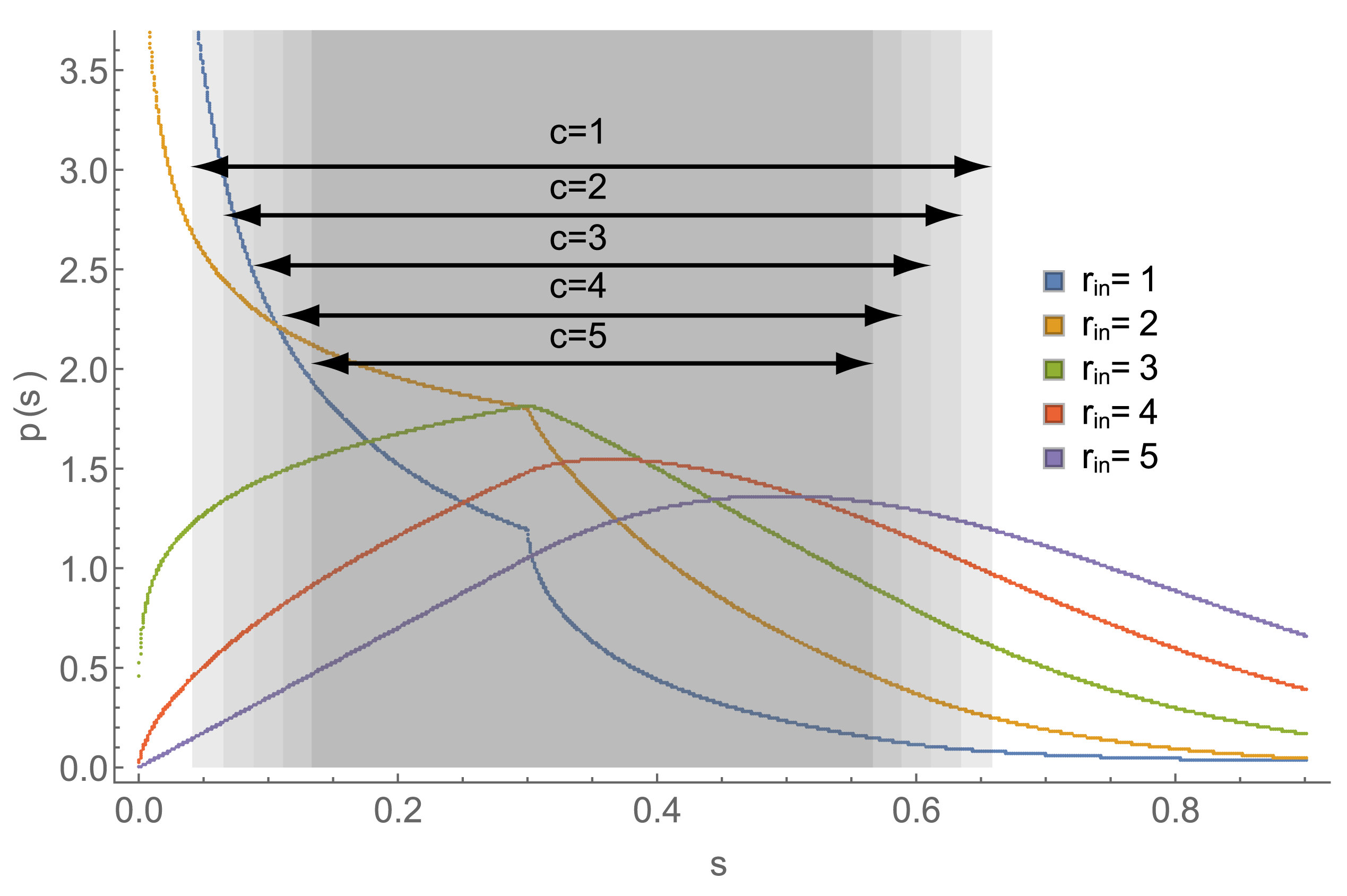

Supplement: S3 Fig — Each curve represents the PDF p of the stimulus function s for different values of incoming rate rin. The gray rectangles represent the size of the oscillatory region in the FN system (b1, b2) for a = 0.35 and different values of volatility c. For all curves, k = 0.3, τ = 0.41. (TIF) [file pcbi.1006200.s003.tif]

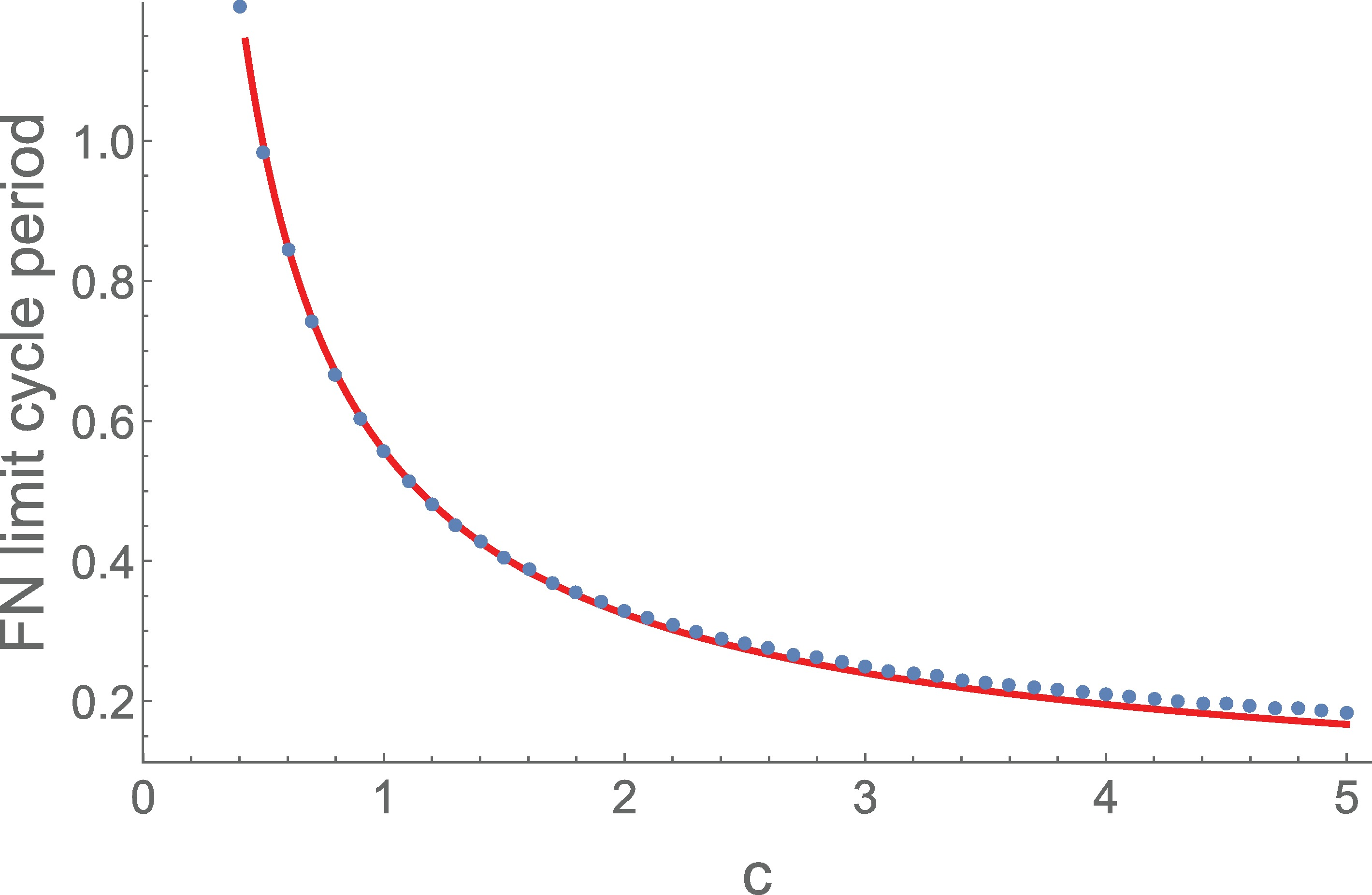

Supplement: S4 Fig — Blue dots represent numerical simulations for the period of the FN limit cycle. The red curve represents the analytical approximation in S1 Text. In both cases we set s = 0.35. (TIF) [file pcbi.1006200.s004.tif]

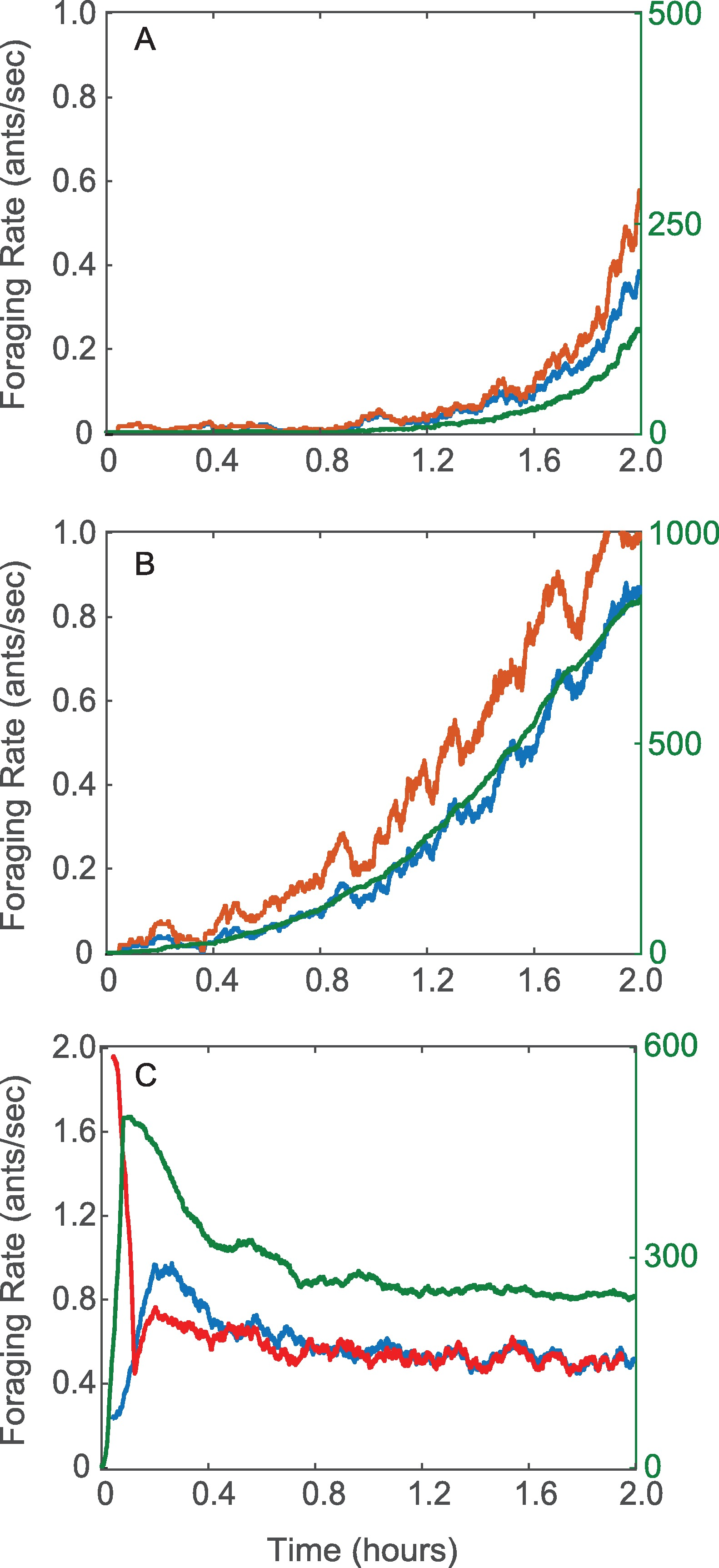

Supplement: S5 Fig — Plots resemble observed foraging behaviors in S2 Fig. Qualitative comparisons can be made between A here and S2C Fig, between B here and S2D Fig, and between C here and in S2E and S2F Fig. A) cu = 0.9, ci = 2.2, N = 500, D = 5. Setting cu < ci where cu is close to c* results in a long period before the rates ramp up. B) cu = 1, ci = 1, N = 1000, D = 15. Setting the mean foraging trip time D to be large results in long lasting transients. C) cu = 0.7, ci = 0.9, N = 1000, D = 7. Setting the initial λin equal to the sequence from the first 5 minutes of λin for Colony 1017 on Aug. 23, 2016 yields the behavior shown in S2E and S2F Fig that follows an initial burst of foragers. (TIF) [file pcbi.1006200.s005.tif]
