# Supplementary material for: Regulation of harvester ant foraging as a closed-loop excitable system
Source: PLoS Comput Biol. 2018 Dec 4;14(12):e1006200. doi: 10.1371/journal.pcbi.1006200 (PMC6294393; doi:10.1371/journal.pcbi.1006200)
Supplement: S1 Table — Average temperature, average relative humidity, temperature at 11 am, and relative humidity at 11 am in Rodeo, New Mexico, USA for days with data plotted in Figs 3 and 4 and S2 Fig. Data collected by the Citizen Weather Observer Program station E8703 and accessed through Weather Underground. The station is located 1.7 miles from the study site. (PDF) [file pcbi.1006200.s006.pdf]

		Daily Average		At 11 am	
**Figure**	**Date**	**Temperature**	**Humidity**	**Temperature**	**Humidity**
3A, 3B	Aug. 20, 2016	25.9C	57%	24.8C	57%
4A, 4B	Aug. 27, 2015	25.3C	58%	27.5C	52%
4C, 4D	Aug. 31, 2015	26.8C	53%	28.8C	45%
4E, 4F	Sept. 1, 2015	25.2C	53%	27.5C	52%
S1A	Sept. 5, 2015	22.6C	77%	23.3C	77%
S1B	Aug. 8, 2016	29.7C	48%	39.9C	43%
S1C	Aug. 20, 2017	23.0C	71%	22.7C	73%
S1D	Aug. 16, 2017	26.0C	48%	27.4C	41%
S1E	Aug. 23, 2016	24.1C	43%	28.8C	36%
S1F	Aug. 18, 2016	25.5C	27%	31.2C	23%

S1 Table. Temperature and relative humidity in Rodeo, New Mexico. Average temperature, average relative humidity, temperature at 11 am, and relative humidity at 11 am in Rodeo, New Mexico, USA for days with data plotted in Fig 3, Fig 4, and S2 Fig. Data collected by the Citizen Weather Observer Program station E8703 and accessed through Weather Underground [1]. The station is located 1.7 miles from the study site.

References

1. The Weather Company LLC, Weather Underground - Ramuda Drive KNMRODEO3;. `https://www.wunderground.com/personal-weather-station/dashboard?ID=KNMRODEO3#history`.
