# Supplementary material for: Regulation of harvester ant foraging as a closed-loop excitable system
Source: PLoS Comput Biol. 2018 Dec 4;14(12):e1006200. doi: 10.1371/journal.pcbi.1006200 (PMC6294393; doi:10.1371/journal.pcbi.1006200)
Supplement: S1 Text — (PDF) [file pcbi.1006200.s007.pdf]

Regulation of Harvester Ant Foraging as a Closed-Loop Excitable System

Renato Pagliara[1], Deborah M. Gordon[2], Naomi Ehrich Leonard[1*],

1 Department of Mechanical and Aerospace Engineering, Princeton University, Princeton, New Jersey, United States of America
2 Department of Biology, Stanford University, Stanford, California, United States of America

*Email: naomi@princeton.edu

S1 Text. Effect of volatility on the frequency of oscillations in the FN.
Here we use results from [1] Chapter III, Theorem 3 to obtain an asymptotic expansion for the period of the limit cycle T_{LC} as ϵ_2 goes to zero. We show that the period of the FN limit cycle is inversely proportional to c by computing the leading term in the expansion.

The limit cycle of the FN is comprised of four components: two fast components that stretch along the v direction between the crest and valley of the cubic nullcline, and two slow components that stretch along the sides of the cubic nullcline. Because it takes much longer to traverse the slow components of the limit cycle than the fast components of the cycle, the period can be approximated by the time it takes trajectories to traverse the two slow components. These slow components run from $v = -2$ to $v = -1$ and from $v = 2$ to $v = 1$, respectively, and are proportional to the length of the sides of the cubic nullcline, which we show are proportional to c.

Theorem 1. *The limit cycle of the FN system*

$$\epsilon_1\epsilon_2 \frac{\mathrm{d}v}{\mathrm{d}t} = v - v^3/3 - cu - 0.35 + s$$
$$\epsilon_1 \frac{\mathrm{d}u}{\mathrm{d}t} = v - cu$$

has the asymptotic representation:

$$T_{LC} = T_0 + C_1\epsilon_2^{2/3} + C_2\epsilon_2 \ln\frac{1}{\epsilon_2} + \mathcal{O}(\epsilon_2),$$

as $\epsilon_2 \to 0$*, where* C_1 *and* C_2 *are constants and where*

$$T_0 = \frac{3\epsilon_1}{c}\left\{\int_{-2}^{-1} \frac{(v^2-1)}{(3z - v^3)}dv + \int_{2}^{1} \frac{(v^2-1)}{(3z - v^3)}dv\right\}.$$

Proof. By Chapter III, Theorem 3 of [1], the limit cycle of the FN model has the asymptotic representation

$$T_{LC} = T_0 + C_1\epsilon_2^{2/3} + C_2\epsilon_2 \ln\frac{1}{\epsilon_2} + C_3\epsilon_2 + \mathcal{O}(\epsilon_2^{4/3}).$$

Or, equivalently,

$$T_{LC} = T_0 + C_1\epsilon_2^{2/3} + C_2\epsilon_2 \ln\frac{1}{\epsilon_2} + \mathcal{O}(\epsilon_2).$$

Let time be scaled by $1/\epsilon_1$ and let $z = s - 0.35$, leading to the new system

$$\epsilon_2 \frac{\mathrm{d}v}{\mathrm{d}t} = v - v^3/3 - cu + z$$
$$\frac{\mathrm{d}u}{\mathrm{d}t} = v - cu$$

The critical manifold of this fast-slow system is $M_0 := \{(v,u) \in \mathbb{R}^2 | u = (v - v^3/3 + z)/c\}$. In the limit $\epsilon_2 \to 0$, the slow manifold is equal to the critical manifold. Let Ψ_0 denote the limit cycle in this limit.

Using the description of M_0 as a graph $u = h(v)$, the dynamics of the system on the slow flow can be written as

$$\frac{\mathrm{d}u}{\mathrm{d}t} = v^3/3 - z.$$

We get a second expression for $\mathrm{d}u/\mathrm{d}t$ by differentiating M_0 with respect to t

$$\frac{\mathrm{d}u}{\mathrm{d}t} = \frac{1-v^2}{c}\frac{\mathrm{d}v}{\mathrm{d}t}.$$

Equating the two expressions we obtain

$$\frac{\mathrm{d}v}{\mathrm{d}t} = \frac{c(v^3-3z)}{3(1-v^2)}.$$

Multiplying both sides by $\mathrm{d}t$ and integrating over Ψ_0,

$$T_0 = \frac{3}{c}\int_{\Psi_0}\frac{(1-v^2)}{(v^3-3z)}\mathrm{d}v.$$

In Ψ_0, the fast components of the orbit take place instantaneously and the time taken to complete the orbit is equal to the time taken to traverse the slow components. The slow components of the trajectory take place on the slow manifold between $v \in [-2,-1]$ and $v \in [1,2]$, yielding the expression

$$T_0 = \frac{3\epsilon_1}{c}\left\{\int_{-2}^{-1}\frac{(1-v^2)}{(v^3-3z)}dv + \int_{2}^{1}\frac{(1-v^2)}{(v^3-3z)}dv\right\}$$

where time has been scaled back to its original form. This expression is inversely proportional to c. Furthermore, this integral has a short closed form solution when the slow components are symmetric (i.e. $z = 0$, or $s = 0.35$),

$$T_0 = 2\,\frac{3\epsilon_1}{c}\int_{-2}^{-1}\frac{(1-v^2)}{v^3}dv = \frac{\epsilon_1}{4c}(-9+8\log 8).$$

We compute the constants C_1 and C_2 by applying formulas 7.9 and 7.10 of [1] Chapter III, Theorem 3. When $s = 0.35$, the flow along the system is symmetric and the constants C_1 and C_2 are given by

$$C_1 = \frac{3.79366\epsilon_1}{c^{1/3}}$$

and

$$C_2 = \frac{-\epsilon_1}{2}.$$

When $s \neq 0.35$, $z \neq 0$ and the flow along the system is not symmetric. In this case the constants C_1 and C_2 can each be represented as the sum of two constants, and the asymptotic representation for T_0 becomes

$$T_{LC} = T_0 + (C_{11}+C_{12})\epsilon_2^{2/3} + (C_{21}+C_{22})\epsilon_2\ln\frac{1}{\epsilon_2} + \mathcal{O}(\epsilon_2)$$

where

$$C_{11} = \frac{3.37214|1-3z|\ \mathrm{Sgn}(1/3-z)}{(-8/9+z(-7/3+z))(c/\epsilon_1^2)^{1/3}},$$

$$C_{12} = \frac{\epsilon_1(-2+6z+|1-3z|)}{2(1-3z)^2},$$

$$C_{21} = \frac{3.37214|1+3z|\ \mathrm{Sgn}(1/3+z)}{(-8/9+z(-7/3+z))(c/\epsilon_1^2)^{1/3}},$$

$$C_{22} = \frac{\epsilon_1(-2-6z+|1+3z|)}{2(1+3z)^2}$$

and Sgn represents the sign function. □

References

1. Mishchenko E, Rozov N Kh. Differential equations with small parameters and relaxation oscillations. Boston: Springer; 1980.
