# Supplementary material for: Regulation of harvester ant foraging as a closed-loop excitable system
Source: PLoS Comput Biol. 2018 Dec 4;14(12):e1006200. doi: 10.1371/journal.pcbi.1006200 (PMC6294393; doi:10.1371/journal.pcbi.1006200)
Supplement: S2 Text — (PDF) [file pcbi.1006200.s008.pdf]

Regulation of Harvester Ant Foraging as a Closed-Loop Excitable System

Renato Pagliara[1], Deborah M. Gordon[2], Naomi Ehrich Leonard[1*],

1 Department of Mechanical and Aerospace Engineering, Princeton University, Princeton, New Jersey, United States of America
2 Department of Biology, Stanford University, Stanford, California, United States of America

*Email: naomi@princeton.edu

S2 Text. Additional field observations of foraging rates.

Here we present additional details for the field observations of foraging rates shown in S2 Fig.

Panel A of S2 Fig shows the foraging rates for Colony 863 on September 5, 2015. The rates increased before reaching a QSS at around 10:30 am. The same colony on September 1, 2015 did not exhibit a QSS and had stopped foraging by 11 am (Fig 4E). These observations are consistent with measurements showing that September 5, 2015 was a particularly cool and humid day while September 1, 2015 was much hotter and drier (see S1 Table).

Panel B of S2 Fig shows the data for Colony D19 on August 8, 2016 (from video recording) and provides an example of a very early cessation of foraging where both outgoing and incoming rates reached zero well before 11:00 am, similar to Colony 863 on September 1, 2015 (Fig 4E). Both August 8, 2016 and September 1, 2015 were very hot and dry days (see S1 Table).

Panel C of S2 Fig show the data for Colony 859 on August 20, 2017 (from manual recording) and provides an example where the initial transient took a long time before ramping up. The initial transient for Colony 859 on August 20, 2017 remained at around 0.01 ants/sec from 10 am to 11:15 am before increasing to about 0.4 ants/sec by 12:30 pm. August 20, 2017 was a cool and humid day (see S1 Table). Colonies might prefer different ranges of temperature and humidity; on cool and humid days, colonies that prefer warmer temperatures might forage at slightly later times of the day than colonies that prefer more cool temperatures.

Panel D of S2 Fig show the data for Colony 1107 on August 16, 2017 (from manual recording) and provides a different example of a slow transient; it took from 8 am to 10:30 am for the foraging rates to increase from 0.3 ants/sec to around 0.9 ants/sec. During this period the number of foragers outside the nest reached almost 2000. In this case August 16, 2017 was not a particularly cool or humid day (see S1 Table). The long transient and large numbers of active foragers suggests that the average time it took a forager to find a seed was long. Long foraging trip times can result in slow transients and high numbers of active foragers because when foragers take a long time to find a seed, it takes longer for foragers to return to the nest and interact with available foragers who then become active foragers. As well, when the average foraging trip time is long, more foragers might be required to cover larger and less dense foraging areas.

Panel E and F of S2 Fig show the data for Colony 1017 on August 23, 2016 (from manual recording) and for Colony 1015 on August 18, 2016 and provide two examples of a burst in the outgoing foraging rate at the start of the foraging day that rapidly increases the number of active foragers outside the nest; it took from 7:30 am to 7:45 am for Colony 1017 on August 23, 2016 to increase the number of active foragers from 0 to 800 and it took from 7:15 am to 7:30 am for Colony 1015 on August 18, 2016 to increase the number of active foragers by 600. In both cases, the foraging rates reached a QSS that lasted tens of minutes. Both August 23 and August 18, 2016 were very dry days (see S1 Table).

The burst kick starts the foraging process very rapidly and appears to be different from the mechanism that activates available foragers to leave the nest through interactions between incoming successful foragers and the available foragers. The rapid increase in the number of active foragers outside the nest might be advantageous on hot and dry days on which there will be only a short period of time in the early morning with acceptable foraging conditions.
