# Supplementary material for: Regulation of harvester ant foraging as a closed-loop excitable system
Source: PLoS Comput Biol. 2018 Dec 4;14(12):e1006200. doi: 10.1371/journal.pcbi.1006200 (PMC6294393; doi:10.1371/journal.pcbi.1006200)
Supplement: S3 Text — (PDF) [file pcbi.1006200.s009.pdf]

Regulation of Harvester Ant Foraging as a Closed-Loop Excitable System

Renato Pagliara[1], Deborah M. Gordon[2], Naomi Ehrich Leonard[1*],

1 Department of Mechanical and Aerospace Engineering, Princeton University, Princeton, New Jersey, United States of America
2 Department of Biology, Stanford University, Stanford, California, United States of America

*Email: naomi@princeton.edu

S3 Text. Analytical Approximation for $\bar{\mathbf{r}}_{\mathbf{out}}$ in terms of $\bar{\mathbf{r}}_{\mathbf{in}}$ and c.

Under the assumption that λ_{in} is a Poisson process with constant rate $\bar{r}_{in}$, Eq. (4) is equivalent to a Poisson shot-noise process with exponential decay:

$$s(t) = s(0)h(t) + \sum_{i=1}^{N(t)} k\ h(t - t_i)$$

where t_i are the jump times of the Poisson process, and

$$h(t) = \begin{cases} e^{-t/\tau}, & t \geq 0 \\ 0, & t < 0. \end{cases}$$

The mean and variance of this random process for an initial condition $s(0) = 0$ are given by $\bar{r}_{in}\tau k(1 - e^{-t/\tau})$ and $\frac{1}{2}\bar{r}_{in}\tau k^2(1 - e^{-2t/\tau})$ respectively [1]. Shot-noise processes are Markovian and it can be shown that for finite jump sizes, $k < \infty$, s is ergodic [2], meaning that as $t \to \infty$, $s(t)$ converges in total variation to a unique stationary probability distribution $\pi(s)$ for any initial condition $s(0)$. In other words, s has the property that time averages converge in time to statistical averages. The ergodicity of s allows us to find an asymptotic expression as $t \to \infty$ for the expected fraction of time that any single outcome of the random process spends in a region (b_1, b_2) by looking at its stationary probability density function.

Let $S_f = \{t_f \in [t_0, t_0 + T] \mid b_1 < s < b_2\}$ be the set of all times over the time interval $[t_0, t_0 + T]$ for which the stimulus is in the (b_1, b_2) region. Then $S_f \subseteq S$ where $S = \{t \in [t_0, t_0 + T]\}$. We define $\mathbb{1}_{S_f} : S \to \{0, 1\}$ to be the indicator function associated with the subset S_f:

$$\mathbb{1}_{S_f}(t) = \begin{cases} 1 & t \in S_f \\ 0 & \text{otherwise.} \end{cases}$$

Let T_f be the amount of time that s is between b_1 and b_2:

$$T_f = \int_{t_0}^{t_0+T} \mathbb{1}_{S_f}(t)\ \mathrm{d}t.$$

From the ergodic properties of s, and by the strong law of large numbers,

$$\lim_{T\to\infty} \frac{1}{T} \int_T \mathbb{1}_{S_f}(s)\ \mathrm{d}s = \int_{b_1}^{b_2} p(s)\ \mathrm{d}s,$$

where $p(s)$ is the density associated with $\pi(s)$, i.e. the stationary probability density function (PDF) of s:

$$\pi(s) = \int_0^s p(y)\ \mathrm{d}y.$$

The PDF (see S4 Text) is given as a piecewise function $p_n(s)$ for $(n-1)k \leq s < nk$ where the piecewise elements satisfy recurrence equations that depend on $\bar{r}_{in}$, τ, and k.

Let b_1 and b_2 be the FN bifurcation values of the input to the FN that takes the system from quiescence into the oscillatory regime and from this regime into saturation respectively:

$$b_{1,2} = 0.35 \mp \frac{1}{3}(1 - c\epsilon_2)^{3/2}.$$

The size of the oscillatory region is given by the difference between b_2 and b_1 and it decreases with increasing volatility c (see S3 Fig). For constant s where $b_1 < s < b_2$, the output rate is a constant given by the oscillation frequency of the FN when driven by a constant input s.

For s not constant, the FN transitions between quiescence, oscillatory behavior, and saturation as s varies. For $\epsilon_1 \ll 1$, the FN dynamics are much faster than the dynamics of s, and the number of foragers leaving the nest in a given time period $[t_0, t_0 + T]$ is proportional to T_f, the amount of time spent by s in the oscillatory region.

For $T \to \infty$, nonlinear effects in the oscillations become negligible and the mean outgoing rate becomes

$$\bar{r}_{out} = \lim_{T\to\infty} \frac{1}{T} \int_T f_{\epsilon 2}(s) \cdot \mathbb{1}_{S_f}(s) \; \mathrm{d}s$$

where $f_{\epsilon 2}$ is the mean oscillation frequency of the FN when the driving input is constant and equal to s. We approximate $f_{\epsilon 2} = 1/T_{LC}$ through the asymptotic representation [3]:

$$T_{LC} = T_0 + C_1 \epsilon_2^{2/3} + C_2 \epsilon_2 \ln \frac{1}{\epsilon_2} + \mathcal{O}(\epsilon_2),$$

where T_0, C_1, and C_2 are given in S1 Text to obtain an approximate expression for how $\bar{r}_{out}$ changes as a function of both $\bar{r}_{in}$ and c:

$$\bar{r}_{out} = \int_{b_1}^{b_2} \frac{p(s, \bar{r}_{in})}{T_{LC}(s, c)} \; \mathrm{d}s.$$

References

1. Ross SM. Stochastic Processes. 2nd ed. Wiley series in mathematical statistics. Probability and mathematical statistics. Wiley; 1996.

2. Borovkov K, Novikov A. On a piece-wise deterministic Markov process model. Stat Probab Lett. 2001;53(4):421–428.

3. Mishchenko E, Rozov N Kh. Differential equations with small parameters and relaxation oscillations. Boston: Springer; 1980.
