# Supplementary material for: Regulation of harvester ant foraging as a closed-loop excitable system
Source: PLoS Comput Biol. 2018 Dec 4;14(12):e1006200. doi: 10.1371/journal.pcbi.1006200 (PMC6294393; doi:10.1371/journal.pcbi.1006200)
Supplement: S4 Text — (PDF) [file pcbi.1006200.s010.pdf]

Regulation of Harvester Ant Foraging as a Closed-Loop Excitable System

Renato Pagliara[1], Deborah M. Gordon[2], Naomi Ehrich Leonard[1*],

1 Department of Mechanical and Aerospace Engineering, Princeton University, Princeton, New Jersey, United States of America
2 Department of Biology, Stanford University, Stanford, California, United States of America

*Email: naomi@princeton.edu

S4 Text. Probability Density Function of $\mathbf{s(t)}$.

Here we find an analytical description of the probability density function of the stimulus function $s(t)$ under the assumption that the incoming rate is a Poisson process. Under this assumption $s(t)$ takes the form of a Poisson shot-noise process. Before we state our results, we state a result by Gilbert and Pollak (1959) [1]:

Lemma 1. *The amplitude distribution function $F_s(\xi) = Pr[s(t) \leq \xi]$ for the Poisson shot-noise process*

$$s(t) = \sum_{i=-\infty}^{N(t)} h(t - t_i),$$

where $h(t)$ is called the impulse shape function, satisfies the integral equation

$$sF_s(s) = \int_{-\infty}^{s} F_s(x)\ dx + \bar{r}_{in} \int_{-\infty}^{\infty} F_s(s - h(t))\ h(t)\ dt$$

where $\bar{r}_{in}$ is the rate parameter of the underlying Poisson process.

Proof. We refer the reader to [1] for the proof. □

Theorem 2. *Consider the shot-noise process with exponential decay and impulses arriving with rate $\bar{r}_{in}$ given by*

$$s(t) = \sum_{i=1}^{N(t)} k\ h(t - t_i),$$

where $k > 0$ is a constant and

$$h(t) = \begin{cases} e^{-t/\tau}, & t \geq 0 \\ 0, & t < 0. \end{cases}$$

Then, the steady-state probability density function of $s(t)$ can be written as a piecewise function $p(s)$ where the piecewise elements $p_n(s)$ for $(n-1)k \leq s < nk$ satisfy the recurrence equations:

$$p_n(s) = p_{n-1}(s) + \alpha s^{\bar{r}_{in}\tau - 1}(-\bar{r}_{in}\tau)^{n-1} g_n(s, \bar{r}_{in}, k, \tau)$$

$$g_n(s, \bar{r}_{in}, k, \tau) = \int_{k}^{s} (x - k)^{\bar{r}_{in}\tau - 1} x^{-\bar{r}_{in}\tau} g_{n-1}(x - k, \bar{r}_{in}, k, \tau) dx$$

with

$$\alpha = \frac{(ke^{\gamma})^{-\bar{r}_{in}\tau}}{\Gamma(\bar{r}_{in}\tau)} s^{\bar{r}_{in}\tau - 1}, \quad p_0(s) = 0, \quad g_1(s, \bar{r}_{in}, k, \tau) = 1,$$

where $\gamma = 0.5772...$ is Euler's constant and Γ is the gamma function.

Proof. For a Poisson shot-noise process with impulse shape function:

$$h(t) = \begin{cases} e^{-t/\tau}, & t \geq 0 \\ 0, & t < 0, \end{cases}$$

the integral equation in Lemma 1 can be rewritten as

$$sp(s) = \bar{r}_{in}\tau \int_0^k p(s-\xi)\ \mathrm{d}\xi = \bar{r}_{in}\tau \int_{s-k}^s p(x)\ \mathrm{d}x,$$

where $p(s) = \mathrm{d}F_s/\mathrm{d}s$ is the density function of s.

Differentiating with respect to s, we obtain

$$s\frac{dp}{ds} + p(s)[1-\bar{r}_{in}\tau] = -\bar{r}_{in}\tau p(s-k). \tag{1}$$

When $0 \leq s \leq k$, then $p(s-k) = 0$ and

$$s\frac{dp}{ds} + p(s)[1-\bar{r}_{in}\tau] = 0.$$

Picking $p(s) = \alpha\ s^{\bar{r}_{in}\tau-1}$ satisfies the equation above. Thus, we have obtained a solution for $p(s) = p_1(s)$ when $0 \leq s \leq k$. For $s > k$, the differential equation Eq. (1) may be converted to an integral form:

$$p(s) = s^{\bar{r}_{in}\tau-1}\left[\alpha - \bar{r}_{in}\tau \int_k^s p(x-k)x^{-\bar{r}_{in}\tau}\ \mathrm{d}x\right]. \tag{2}$$

Since the integrand is known for $k < x < 2k$, we can determine $p(s) = p_2(s)$ for $k < s < 2k$. This in turn enables us to integrate further to get $p(s) = p_3(s)$ for $2k < s < 3k$, etc. Let $m = \bar{r}_{in}\tau$, then the results for the first three jump regions $p_n(s)$, $n = 1, 2, 3$ are given by

$$\begin{aligned}
p_1(s) &= \alpha\ s^{m-1} \\
p_2(s) &= p_1(s) - \alpha\ s^{m-1}m\int_k^s (x-k)^{m-1}x^{-m}\ \mathrm{d}x \\
p_3(s) &= p_2(s) + \alpha\ s^{m-1}m^2\int_k^s (x-k)^{m-1}x^{-m}\int_k^{x-k}(\xi-k)^{m-1}\xi^{-m}\mathrm{d}\xi\ \mathrm{d}x.
\end{aligned}$$

We now show by induction that $p(s) = p_n(s)$, for $(n-1)k \leq s < nk$, satisfies the following recurrence equations:

$$\begin{aligned}
p_n(s) &= p_{n-1}(s) + \alpha s^{m-1}(-m)^{n-1}g_n(s) \\
g_n(s) &= \int_k^s (x-k)^{m-1}x^{-m}g_{n-1}(x-k)\ \mathrm{d}x
\end{aligned}$$

with $p_0 = 0$, $g_1(s) = 1$. For $n = 1$,

$$p_1(s) = p_0(s) + \alpha s^{m-1}(-m)^0 g_1(s) = \alpha s^{m-1}$$

as expected. Now, assume that for $n = j$,

$$\begin{aligned}
p_j(s) &= p_{j-1}(s) + \alpha s^{m-1}(-m)^{j-1}g_j(s) \\
g_j(s) &= \int_k^s (x-k)^{m-1}x^{-m}g_{j-1}(x-k)\ \mathrm{d}x.
\end{aligned}$$

Then, for $n = j+1$,

$$\begin{aligned}
p_{j+1}(s) &= s^{m-1}\left[\alpha - m\int_k^s p_j(x-k)x^{-m}\ \mathrm{d}x\right] \\
&= s^{m-1}\Bigg[\alpha - m\int_k^s x^{-m}p_{j-1}(x-k)\ \mathrm{d}x \\
&\quad + \alpha(-m)^j\int_k^s (x-k)^{m-1}x^{-m}g_j(x-k)\ \mathrm{d}x\Bigg] \\
&= s^{m-1}\left[\alpha - m\int_k^s x^{-m}p_{j-1}(x-k)\ \mathrm{d}x\right] + \alpha s^{m-1}(-m)^j g_{j+1}(s) \\
&= p_j(s) + \alpha s^{m-1}(-m)^j g_{j+1}(s).
\end{aligned}$$

Finally, the constant α must be determined by the condition

$$\int_0^\infty p(s)\ \mathrm{d}s = 1.$$

To compute the constant, we first note that the characteristic equation of s is given by

$$C(\zeta) = \exp\left[-\bar{r}_{in} \int_{-\infty}^{\infty} (1 - \exp[-\zeta F_s(t)])\ \mathrm{d}t\right]$$

(see [2] for derivation). The characteristic function is the Laplace transform $\hat{p}$ of p,

$$\hat{p}(\zeta) = \exp\left[-\bar{r}_{in}\tau \int_0^{\zeta k} \frac{1 - e^{-y}}{y}\ \mathrm{d}y\right].$$

Using partial integration, this can be rewritten as

$$\begin{aligned}\hat{p}(\zeta) &= \exp\left[-\bar{r}_{in}\tau(1 - e^{-\zeta k})\log \zeta k + \bar{r}_{in}\tau\left(\int_0^\infty e^{-y}\log y\ \mathrm{d}y - \int_s^\infty e^{-y}\log y\ \mathrm{d}y\right)\right]\\ &= (\zeta k)^{-\bar{r}_{in}\tau} e^{-\bar{r}_{in}\tau\gamma}(1 + \mathcal{O}[e^{-\zeta k(1-\varepsilon)}]) \quad \text{for any } \varepsilon > 0.\end{aligned}$$

Thus, for $0 \le s \le k$,

$$\alpha = \frac{(ke^{\gamma})^{-\bar{r}_{in}\tau}}{\Gamma(\bar{r}_{in}\tau)} s^{\bar{r}_{in}\tau - 1}$$

where $\gamma = 0.5772...$ is Euler's constant and Γ is the gamma function. □

References

1. Gilbert E, Pollak H. Amplitude distribution of shot noise. Bell Syst Tech J. 1960;39(2):333–350.

2. Ross SM. Stochastic Processes. 2nd ed. Wiley series in mathematical statistics. Probability and mathematical statistics. Wiley; 1996.
